# Transcriptome profiling of microRNAs reveals potential mechanisms of manual therapy alleviating neuropathic pain through microRNA-547-3p-mediated Map4k4/NF-κb signaling pathway

**DOI:** 10.1186/s12974-022-02568-x

**Published:** 2022-09-01

**Authors:** Chongjie Yao, Jun Ren, Ruixin Huang, Cheng Tang, Yanbin Cheng, Zhizhen Lv, Lingjun Kong, Sitong Fang, Jiming Tao, Yangyang Fu, Qingguang Zhu, Min Fang

**Affiliations:** 1 Yueyang Hospital of Integrated Traditional Chinese and Western Medicine, Shanghai University of Traditional Chinese Medicine, Shanghai, 200437 People’s Republic of China; 2 Research Institute of Tuina, Shanghai Academy of Traditional Chinese Medicine, Shanghai, 200437 People’s Republic of China; 3 School of Acupuncture-Moxibustion and Tuina, Shanghai University of Traditional Chinese Medicine, Shanghai, 201203 People’s Republic of China; 4 The Third Clinical Medical College, Zhejiang Chinese Medical University, Hangzhou, 310053 People’s Republic of China; 5 Shuguang Hospital, Shanghai University of Traditional Chinese Medicine, Shanghai, 201203 People’s Republic of China

**Keywords:** Neuropathic pain, Manual therapy, MiRNA, Lumbar disk herniation, Inflammation, MicroRNA-547-3p, Map4k4

## Abstract

**Background:**

Local neuroinflammation secondary to spinal nerve compression in lumbar disk herniation (LDH) is a key driver contributing to neuropathic pain. Manual therapy (MT), a widely used nonsurgical therapy, can relieve LDH-mediated pain by reducing inflammation. MT has attracted extensive attention; however, its mechanism remains poorly understood. MicroRNAs (miRNAs) are important regulators of pain signaling transduction, but are rarely reported in the chronic compression of dorsal root ganglia (CCD) model, and further investigation is needed to decipher whether they mediate anti-inflammatory and analgesic effects of MT.

**Methods:**

We used a combination of in vivo behavioral and molecular techniques to study MT intervention mechanisms. Neuropathic pain was induced in a CCD rat model and MT intervention was performed according to standard procedures. Enzyme-linked immunosorbent assay (ELISA) was used to detect inflammatory cytokine levels in dorsal root ganglia (DRG). Small RNA sequencing, immunofluorescence, Western blot, and qRT-PCR were performed to screen miRNAs and their target genes and determine core factors in the pathway possibly regulated by miRNA-mediated target gene in DRG of MT-treated CCD rats.

**Results:**

Compared with naive rats, small RNA sequencing detected 22 differentially expressed miRNAs in DRG of CCD rats, and compared with CCD rats, MT-treated rats presented 19 differentially expressed miRNAs, which were functionally associated with nerve injury and inflammation. Among these, miR-547-3p was screened as a key miRNA mediating neuroinflammation and participating in neuropathic pain. We confirmed in vitro that its function is achieved by directly regulating its target gene Map4k4. Intrathecal injection of miR-547-3p agomir or MT intervention significantly reduced Map4k4 expression and the expression and phosphorylation of IκBα and p65 in the NF-κB pathway, thus reducing the inflammatory cytokine levels and exerting an analgesic effect, whereas intrathecal injection of miR-547-3p antagomir led to opposite effects.

**Conclusions:**

In rats, CCD-induced neuropathic pain leads to variation in miRNA expression in DRG, and MT can intervene the transcription and translation of inflammation-related genes through miRNAs to improve neuroinflammation and alleviate neuropathic pain. MiR-547-3p may be a key target of MT for anti-inflammatory and analgesia effects, which is achieved by mediating the Map4k4/NF-κB pathway to regulate downstream inflammatory cytokines.

**Supplementary Information:**

The online version contains supplementary material available at 10.1186/s12974-022-02568-x.

## Background

Lumbar disk herniation (LDH) is a series of syndromes characterized by low back and radiating pain in the lower extremities due to degeneration or injury of the lumbar disk. This results in the rupture of the annulus fibrosus and herniation of the nucleus pulposus beyond the normal limits of the intervertebral disk space, which irritates or compresses the spinal nerve root [1]. With the changing lifestyle of modern people, LDH incidence has increased significantly, and neuropathic pain caused by nerve injury is now the main clinical symptom of LDH. Thus, LDH has become an important public health issue, posing a huge burden on countries, societies, and families [2].

Although several studies have focused on LDH-triggered pain [3–5], current treatments are not fully effective in controlling such neuropathic pain, notably because our understanding of the mechanisms that underlying the pain is incomplete [6]. Recent research has found that the degree of pain caused by LDH is not completely consistent with the actual situation shown on imaging [7], and several patients with lumbar disk herniation do not experience obvious pain sensation, or the site of pain is not the lesion segment. Regarding the inflammatory response associated with pain, inflammatory receptors in dorsal root ganglion (DRG) neurons can be activated to provoke pain and hyperalgesia when experimenters induce radicular inflammation in animal models [8]. Therefore, the generation of an inflammatory microenvironment in DRG may be a key driver during the development of LDH-induced neuropathic pain.

Manual therapy (MT), also known as Tuina in China, has become a commonly used method for pain relief in clinics [9, 10], and some researchers believe that its efficacy in LDH is superior to other conventional nonsurgical therapies, such as acupuncture and traction [11]. It is widely believed that mechanical allodynia and hyperalgesia have been associated with the increasing expression of proinflammatory cytokines such as tumor necrosis factor (TNF)-α, interleukin (IL)-1β, and IL-6 [12]. Studies on analgesia [13, 14] have confirmed that MT can significantly improve the inflammatory response to pain in local tissues of the body by reducing the levels of inflammatory cytokines. However, the anti-inflammatory and analgesic mechanisms of MT remain poorly understood.

With the advancement of recent pain-related research, studies have found that non-coding RNAs are important regulators of cellular homeostasis [15], including microRNAs (miRNAs) [16]. The dysregulation of their expression is closely associated with several disorders, such as cancer [17], cardiovascular [18], and neurodegenerative diseases [19]. MiRNAs have specific regulatory functions in cells and can inhibit the expression of neural function-related target genes, thus exerting effects on pain signal transduction [20]. However, the role of miRNAs in neuropathic pain caused by nerve root compression has not been fully investigated, and whether they mediate MT analgesic effect has not yet been demonstrated.

As a traditional nonpharmacological therapy, the multipathways and multitarget characteristics of MT are highly consistent with the modes of action of miRNAs [21]. RNA sequencing technology has revealed that MT can regulate gene expression of tissues and promote repair and regeneration following peripheral nerve injury, thus confirming that MT can intervene the pain caused by DRG injury at a transcriptomic level. According to this study, it can be hypothesized that the improvement in inflammation and pain due to MT is partly achieved by miRNA regulation and that MT affects its target genes by regulating key miRNAs, thus playing anti-inflammatory and analgesic roles.

Therefore, this study aimed to confirm that MT can effectively alleviate the inflammation response induced by LDH by regulating specific miRNAs, consequently inhibiting the incidence and maintenance of neuropathic pain. In our study, miR-547-3p was screened as a key miRNA for MT in pain relief. We demonstrated that MT can improve the inflammatory microenvironment in DRG by inhibiting the activation of the nuclear factor kappa-B (NF-κB) signaling pathway via its target gene Map4k4. By investigating the regulation of miRNA-547-3p-mediated Map4k4/NF-κb signaling pathway, we provided an experimental and theoretical basis for potential MT anti-inflammatory and analgesic mechanisms.

## Materials and methods

### Animals

Male Sprague–Dawley rats weighing 200–240 g were obtained from Shanghai Jihui Laboratory Animal Co., Ltd. (Shanghai, China) and housed in the Laboratory Animal Center of Yueyang Hospital of Integrated Traditional Chinese and Western Medicine. All rats were housed under standard laboratory conditions (22–27 °C, 50–70% indoor humidity) under a 12-h light–dark cycle and fed with rat chow and water ad libitum for 1 week before the experiment. All experiments were conducted in accordance with procedures approved by the Experimental Animal Ethics Committee of Yueyang Hospital of Integrated Traditional Chinese and Western Medicine (YYLAC-2021-099).

### Model establishment

According to previously described methods [22–24], chronic compression of dorsal root ganglion (CCD) was performed on rats under aseptic conditions. Briefly, rats were anesthetized via intraperitoneal injection of sodium pentobarbital (Sigma-Aldrich, St. Louis, MO, USA) at a dose of 40 mg/kg, and the paraspinal muscles were blunt separated from the right side to expose the intervertebral foramina of L4 and L5. Two L-shaped stainless-steel rods with a length of 4 mm and a diameter of 0.6 mm were implanted into the intervertebral foramina of L4 and L5, respectively, at an angle of 30° with the spinal column to generate stable pressure on the DRG. After surgery, muscles, fascia, and skin of the rats were sutured, and erythromycin ointment was applied to prevent infection.

After surgery, the rats were examined via X-ray imaging (Samsung XGEO GU60, Seoul, South Korea) to verify the success of the CCD surgery. Animals with unfixed rods or those exhibiting sensory deficits or disabilities after awakening were excluded from the study. Rats in the sham group underwent the same procedure without steel rod insertion, and rats in the naive group were only anesthetized.

### Manual therapy intervention

**Fig. 1:**
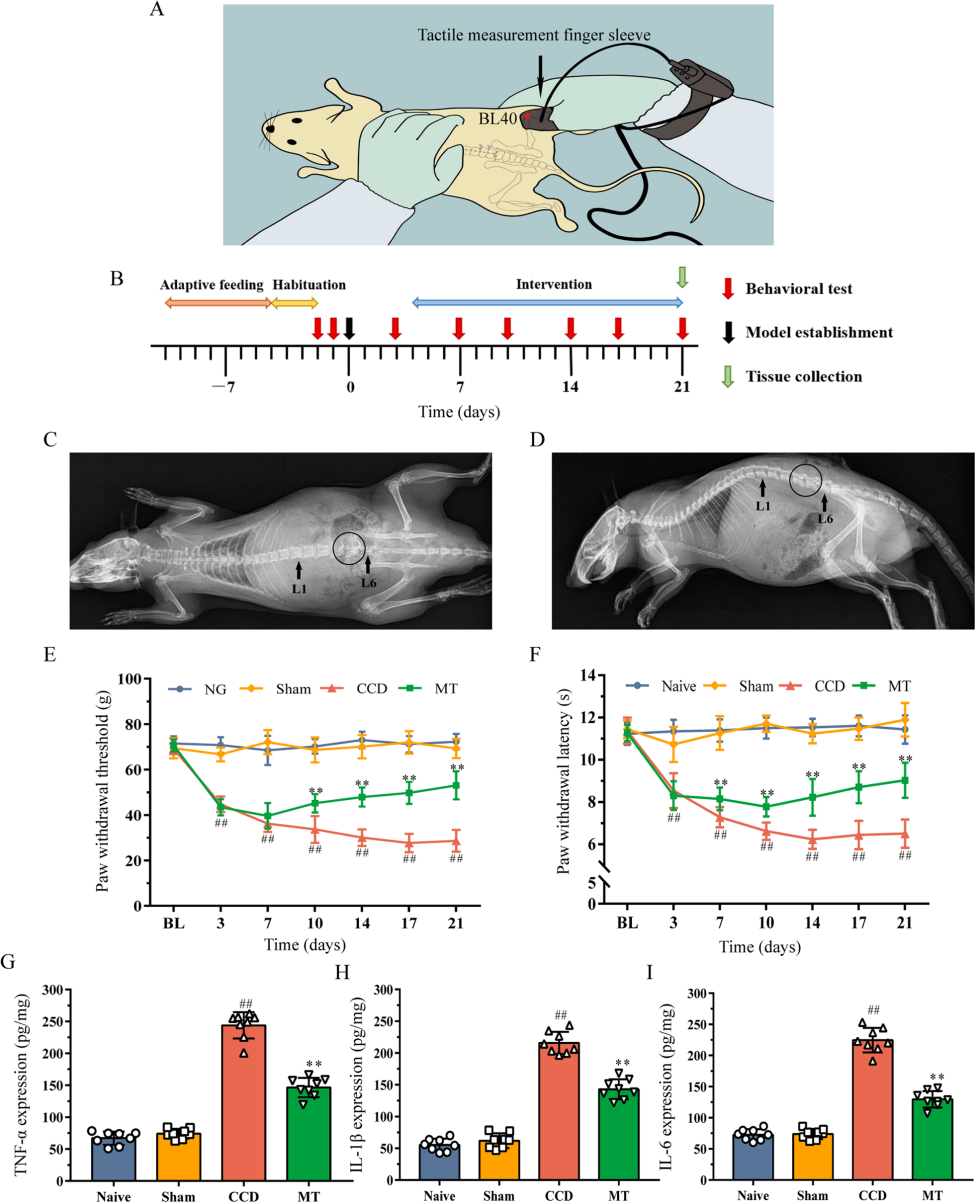
Effects of MT on behavior and inflammatory cytokines in rats DRG. **A** Diagram of MT intervention. **B** Timeline of the experiment. **C**, **D** Radiographs presenting coronal and sagittal views of a rat after CCD surgery. Arrows indicate L1 and L6 to locate the lumbar spine, and circles indicate the L4 and L5 intervertebral foramen where the stainless-steel rod has been implanted. **E**, **F** PWT and PWL of rats were measured to assess their mechanical and thermal hyperalgesia. The data are expressed as mean ± SD (*N* = 8). Repeated measurement ANOVA was used to analyze the data at various time points between the groups and one-way ANOVA followed by LSD post hoc analysis was used for pairwise comparison (^##^*P* < 0.01 versus sham group, ^**^P < 0.01 versus CCD group). **G**–**I** ELISA experiments showed that the expression of TNF-α, IL-1β, and IL-6 in DRG varies between the groups. The data are expressed as mean ± SD (*N* = 8) and analyzed for statistical significance using one-way ANOVA followed by LSD post hoc analysis for multiple comparisons (^##^*P* < 0.01 versus sham group, ^**^P < 0.01 versus CCD group). *ANOVA* analysis of variance, *CCD* chronic compression of dorsal root ganglion, *DRG* dorsal root ganglion, *ELISA* enzyme-linked immunosorbent assay, *LSD* least significant difference, *MT* manual therapy, *PWL* paw withdrawal latency, *PWT* paw withdrawal threshold, *SD* standard deviation

Half of the rats in the CCD group were randomly classified for MT intervention. Based on our previous studies [25–27], specific MT intervention was performed as follows: (1) the intervention started on the 4th day after surgery and was performed once a day for 10 min for 18 consecutive days; (2) rats were handled by the experimenters to adapt 30 min before the intervention; (3) the right thumb of the experimenter was used to press and knead the “Weizhong” (BL40) point on the right lower extremity of the rat, which is located in the center of the crease of the popliteal fossa [28] (Fig. 1A); (4) the right thumb was equipped with a tactile measurement finger sleeve (FingerTPS, Pressure Profile System, CA, USA) to maintain a constant stimulation pressure of 5 N and frequency of 2 Hz. Groups that did not receive MT performed daily grasping in the same manner as the MT group to eliminate potential stress effect.

### Behavioral test

Behavioral tests were conducted during the light cycle, i.e., between 9 am and 5 pm, once a day on 2 days before CCD surgery to measure the baseline, and then on days 3, 7, 10, 14, 17, and 21 after surgery. Before testing, the rats were habituated to the experimental environment for 3 days, 2 h daily. The tests were implemented and recorded by two experimenters who were blinded to the rat groups [29].

An electric von Frey esthesiometer (ALMEMO 2450, IITC/Life Science, Woodland Hills, CA, USA) was applied to evaluate the mechanical hyperalgesia of the rats [30]. The tip head was aimed at the central part of the right hind foot of the rat and raised at a constant speed of 0.5 cm/s for stimulation. When the esthesiometer recorded the maximum amount of stimulation, which caused the rat to withdraw, the value of this force was assigned to the paw withdrawal threshold (PWT). To prevent sensory sensitization caused by frequent stimulation, the next stimulation was conducted at least 20 s after the previous measurement.

A thermal analgesia tester (Model 390, IITC/Life Science) was applied to test the thermal hyperalgesia of the rats [15]. Each rat was placed individually on a transparent glass surface. The light source was located below the glass and the intensity was set at 65 °C. Radiant light was focused on the central part of the right hind foot of the rat. The paw withdrawal latency (PWL) was defined as the time from the onset of radiant heat to the withdrawal of the hind paw. The radiant heat source was adjusted to result in a baseline of 10–12 s, and a cutoff time of 20 s was set to avoid tissue damage. To prevent sensory sensitization caused by frequent stimulation, the next stimulation was conducted at least 10 min after the previous measurement.

In accordance with the method previously described, each rat repeated the test 5 times. The maximum and minimum of the five values were removed, and the mean of the remaining three values was deemed as the result of a test. At the end of behavior testing, L4 and L5 DRG on the right side were dissected for subsequent experimental detection.

### Enzyme-linked immunosorbent assay

DRG were collected, homogenized, and lysed thoroughly in radioimmunoprecipitation assay Lysis Buffer (Beyotime, Shanghai, China) at a concentration of 10 μl/mg. The cell lysate was centrifuged at 12,000×*g* for 5 min at 4 °C, and the protein concentration of the supernatant was calculated using a bicinchoninic acid assay kit (Beyotime). The TNF-α, IL-1β, and IL-6 levels in the supernatant were detected using enzyme-linked immunosorbent assay (ELISA) kits (BOSTER, Wuhan, Hubei, China) according to the manufacturer’s protocol, with each sample tested in duplicates.

### Total RNA extraction and small RNA sequencing

Total RNA was extracted from fresh DRG using RNAiso Plus (TaKaRa Bio, Shiga, Japan). RNA quality was controlled using Nano Drop (Thermo Fisher Scientific, Waltham, MA, USA) and Agilent 2200 TapeStation (Agilent Technologies, Santa Clara, CA, USA). Approximately 1 μg of total RNA per sample was used to construct the libraries. RNA fragments with a length of 18–30 nt were obtained via electrophoretic gel cutting method, reverse transcribed into cDNA, and then amplified via real-time quantitative polymerase chain reaction (qRT-PCR). Target fragments were recovered and rechecked. Qualified libraries were sequenced using Hiseq 2500 (Illumina, San Diego, CA, USA).

### Bioinformatics analysis

High-quality clean reads were obtained using primary sequencing by removing splice sequence, low-quality reads, and reads with fragment lengths of < 17 nt. Clean reads were mapped to the reference genome in the miRBase (https://www.mirbase.org/) using the miRDeep2 software [31] to particularly obtain the structure, length, and expression of miRNAs. The degree of difference between the samples was reflected by fold change (FC) and *P* values of the variation in miRNA expression between different groups were calculated using DESeq (Bioconductor, https://www.bioconductor.org/packages/2.10/bioc/html/DESeq.html). [32]. The criterion for differentially expressed miRNA was the absolute value of log_2_FC > 1 and *P* < 0.05. Target genes of differentially expressed miRNAs between the groups were predicted using TargetScan (https://www.targetscan.org/), miRDB (http://mirdb.org/), miRTarBase (http://miRTarBase.cuhk.edu.cn/), and miRWalk (http://mirwalk.umm.uni-heidelberg.de/). The common results predicted by mirRDB, miRWalk, and TargetScan were combined with those of miRTarBase to obtain the most reliable number of target genes. Functional annotation of these genes was performed using Gene Ontology (GO) analysis (http://geneontology.org/) to classify them into different hierarchical categories based on molecular function, cellular component, and biological process. In addition, pathway analysis of genes was performed according to the Kyoto Encyclopedia of Genes and Genomes (KEGG) database (http://www.genome.jp/kegg/), which were classified into environmental information processing, human diseases, metabolism, and organismal systems.

### Quantitative real-time polymerase chain reaction

Total RNA was extracted using RNAiso Plus (TaKaRa Bio), and reverse transcription was performed using a PrimeScript RT reagent kit with gDNA Eraser (TaKaRa). Quantitative real-time polymerase chain reaction (qRT-PCR) was performed on a PCR System (Bio-Rad, Hercules, CA, USA) with SYBR Premix Ex Taq (TaKaRa). The reaction conditions were a single cycle at 95 °C for 5 min and 40 cycles at 95 °C for 15 s and 60 °C for 30 s. U6 and GAPDH were used as the reference for data normalization of the miRNA and protein levels, respectively. Each sample was tested in triplicates. The primers used in the study are listed in Table 1. The relative expression was determined using the 2^−ΔΔCt^ method.

**Table 1:** Primer sequences used for qRT-PCR analysis

Gene name	Primer sequence (5′–3′)
miR-375-3p	RT: GTCGTATCCAGTGCAGGGTCCGAGGTATTCGCACTGGATACGATCACGC
	Forward: GCGCTTTGTTCGTTCGGCTC
	Reverse: TGCAGGGTCCGAGGTAT
miR-449a-5p	RT: GTCGTATCCAGTGCAGGGTCCGAGGTATTCGCACTGGATACGAACCAGC
	Forward: GCGCTGGCAGTGTATTGTTA
	Reverse: TGCAGGGTCCGAGGTAT
miR-547-3p	RT: GTCGTATCCAGTGCAGGGTCCGAGGTATTCGCACTGGATACGATCTCAC
	Forward: GCGCATTGGTACTTCTTTAA
	Reverse: TGCAGGGTCCGAGGTAT
U6	Forward: CTCGCTTCGGCAGCACA
	Reverse: AACGCTTCACGAATTTGCGT
Map4k4	Forward: GTCCTGTCCCGTCGAGATTC
	Reverse: GCCAACGCAGTCAAGTCAAT
p65	Forward: GCTTCATATGCGGGCAACAG
	Reverse: AAGCAATGAGCCACTCCCTC
IκBα	Forward: AGACTCGTTCCTGCACTTGG
	Reverse: TCTCGGAGCTCAGGATCACA
GAPDH	Forward: AGGTGACCGCATCTTCTTGT
	Reverse: TACGGCCAAATCCGTTCACA

### Cell culture and transfection

Human embryonic kidney (HEK) 293 T cells (ATCC, Manassas, VA, USA) were cultured in DMEM with 10% fetal bovine serum and 0.5% penicillin/streptomycin at 37 °C and 5% CO_2_. When cells attained > 80% confluence, they were transfected with miR-547-3p mimics or scrambled miRNAs using Lipofectamine 2000 (Invitrogen, Waltham, Massachusetts, USA) according to the manufacturer’s instructions, 48 h before luciferase assay.

Bilateral L4–L5 DRG tissues were dissected from healthy rats under sterile conditions, digested in 0.25% trypsin solution at 37 °C for 15 min after complete denudation of their capsule. When the tissue pieces were flocculent, 2 ml of DMEM/F12 containing 10% fetal bovine serum and 1% penicillin/streptomycin was added to terminate digestion, and a pipette was used to gently blow at least 15 times to prepare a homogeneous cell suspension. DRG neurons were seeded into culture flasks and incubated in a 5% CO_2_ incubator at 37 °C. The culture medium was replaced with Neurobasal-A from the 2nd day, and the culture was completed on the 7th day. The morphology of DRG neurons was observed microscopically and identified via immunofluorescence staining using labeled neuron-specific enolase (Additional file 1). Namipo (Transheep, Shanghai, China) transfection reagent was mixed with miR-547-3p mimics, inhibitor, or scrambled miRNA, and incubated at room temperature for 10 min. The complex was added to DRG neurons for transfection. Transfected and untreated cells (control group) were cultured for 48 h before performing Western blot and qRT-PCR. The sequences of miRNA mimics, miRNA inhibitor, and scrambled miRNA are shown in Additional file 2.

### Vector construction and luciferase reporter gene assay

The target gene for miR-547-3p and binding sites were predicted using the algorithms of TargetScan. Approximately 300 bp sequences of Map4k4-3′ untranslated region (UTR) containing the predicted miR-547-3p binding sites and its mutant sequence synthesized using General Biosystems Co., Ltd. (Chuzhou, Anhui, China) were inserted into a dual-reporter psiCHECK2 plasmid digested by Xhol and NotI. All sequences were verified via sequencing. Luciferase assay was performed 48 h post-transfection using a dual luciferase reporter gene assay kit (Beyotime, China) according to the manufacturer’s protocol. The luciferase activity was measured using SpectraMax M3 (Molecular Devices, Silicon Valley, CA, USA) and was normalized to the Renilla luciferase activity. Each sample was tested in triplicates. The sequences of Map4k4 wild-type (WT) and mutant (MUT) are shown in Additional file 2.

### Intrathecal injection

The miR-547-3p agomir, antagomir, and corresponding negative control used for intrathecal injection were obtained from RiboBio (Guangzhou, Guangdong, China) [33]. According to a previously described method [34, 35], the hair on the waist of rats was removed, the skin was disinfected with Iodophor, and rats were anesthetized with 4% isoflurane (Roward, Shenzhen, Guangdong, China) and maintained at a concentration of 2.5%. Lumbar puncture was performed by gently grasping the iliac crest of the rats and vertically inserting a 10-μl Hamilton microsyringe with a 30-gage needle into the intervertebral space between L4 and L5. A successful puncture was indicated by a tail shiver or flick. The liquid was injected evenly and slowly in approximately 15 s. According to the manufacturer’s instructions, 10 μl of saline containing 10 nmol of drugs was used for injection. Intrathecal injections were administered 6 times, twice a week from the 4th day after surgery.

### Western blot

Samples were extracted for total protein, and the protein concentrations were determined for adjusting. Tissue or cell lysates were separated via electrophoresis on SDS-PAGE gel (Beyotime) and transferred to a PVDF membrane. The membrane was incubated with primary antibodies (Map4k4, 1:1000, Proteintech, 55247-1-AP, Wuhan, Hubei, China; p65, 1:1000, CST, 8242, Danfoss, MA, USA; p-p65, 1:1000, CST, 3033; IκBα, 1:1000, CST, 4812; p-IκBα, 1:1000, CST, 2859; GAPDH, 1:5000, CST, 5174) diluted in PBS overnight at 4 °C after blocking with 5% BSA. The membrane was rinsed thrice with TBST and incubated with secondary antibody (1:5000, Sangon Biotech, Shanghai, China) at room temperature for 2 h. Results were acquired using the ChemiDoc MP system (Bio-Rad) with an enhanced chemiluminescence (ECL) kit (Beyotime) and analyzed using ImageJ.

### Immunofluorescence

L4 and L5 DRG on the right side of the rats were collected and fixed in 4% formaldehyde for 24 h and were then dehydrated in gradient alcohol and embedded in paraffin. After deparaffinization and rehydration, 4-μm sections were treated with citric acid buffer (0.01 mol/L, pH 6.0) at 98° C for 15 min and cooled to room temperature for 1 h for antigen retrieval. They were then incubated in 1% hydrogen peroxide solution for 10 min to block endogenous peroxidase activity. They were further blocked in 5% BSA (Beyotime) at room temperature for 30 min, labeled by primary antibodies (Map4k4, 1:1000, Proteintech, 55247-1-AP), and incubated overnight at 4 °C. After 12 h, the sections were rewarmed at 37 °C for 45 min and incubated with Dylight 488 goat antimouse IgG (1:100, Sangon Biotech) and Alexa Fluor 594-conjugated isolectin B4 (IB4; 1:100, Invitrogen) at room temperature for 1 h in the dark. Nuclei were stained with DAPI (Beyotime) for 2 min. Three fields were randomly selected from each section to calculate the average fluorescence intensity of the target protein under a microscope (BX53, Olympus, Tokyo, Japan) at 400 × magnification using ImageProPlus software.

### Statistical analysis

SPSS 21.0 software (IBM, Chicago, IL, USA) was used for statistical analysis. Normality and homogeneity of variance tests were performed. Data are presented as mean ± standard deviation (SD) if they are normally distributed. If the data conformed to the normal distribution, the comparison of differences among the groups was performed using one-way analysis of variance (ANOVA). If the variances were homogeneous, a pairwise comparison was performed using the least significant difference (LSD) test; if the variances were not homogenous, Dunnett’s T3 test was used for pairwise comparison. The repeated measurement ANOVA was used to analyze the data on the same observation index at various time points between the groups. If the results were statistically different, a multivariate ANOVA was used for pairwise comparison. The significance level of the statistical examination was *α* = 0.05, and *P* < 0.05 was considered statistically significant.

## Results

### Improvement in CCD-induced neuropathic pain via manual therapy

To verify the correct establishment of the CCD model, X-rays of the coronal and sagittal planes of the rat lumbar spine were acquired. Considering the rat iliac spine as the bone marker, it can be used to locate the L6 level through the connection of the highest points on both sides. As shown in Fig. 1C, D, the stainless-steel rods were accurately implanted into the L4 and L5 intervertebral foramen, thus generating stable pressure on the corresponding DRG to induce neuropathic pain.

To assess the effects of MT on mechanical and thermal pain thresholds, PWT and PWL of rats in each group were measured (Fig. 1E, F). Compared with the naive group, PWT and PWL in the sham group decreased slightly on the 3rd day after surgery, but the difference was not statistically significant (*P* > 0.05). They were maintained at the baseline level with the naive group in the following 18 days. PWT and PWL in the CCD group decreased continuously from day 0 to day 14 and were stable in the following days; however, they were significantly lower than those in the sham group at the same time point (*P* < 0.01). After MT intervention, PWT and PWL of rats showed an upward trend, which was significantly higher than those of the CCD group at the same time point from the 10th and 7th day after surgery (*P* < 0.01). Besides, the results of ELISA showed that the expression of TNF-α, IL-1β, and IL-6 in the DRG of rats that underwent CCD surgery was significantly increased compared with the expression observed in the sham group (*P* < 0.01), which could be significantly reduced by MT (*P* < 0.01; Fig. 1G–I).

Therefore, these results showed that mechanical pain and thermal hyperalgesia are observed in rats after CCD surgery, accompanied by increased inflammatory cytokine levels in DRGs. MT intervention can significantly improve these conditions, suggesting that it alleviates CCD-induced neuropathic pain through anti-inflammatory responses.

### Analysis of differentially expressed miRNAs in DRG between the groups

To analyze the differentially expressed miRNAs in DRG between the groups, small RNA sequencing was performed. An inspection of the results indicated high quality of the samples (Additional file 3). After removing low-quality reads from the raw reads, clean reads were obtained, which were mapped to the genome. The absolute values of log_2_FC > 1 and *P* < 0.05 were used as the criteria for differential expression of miRNA.

**Fig. 2:**
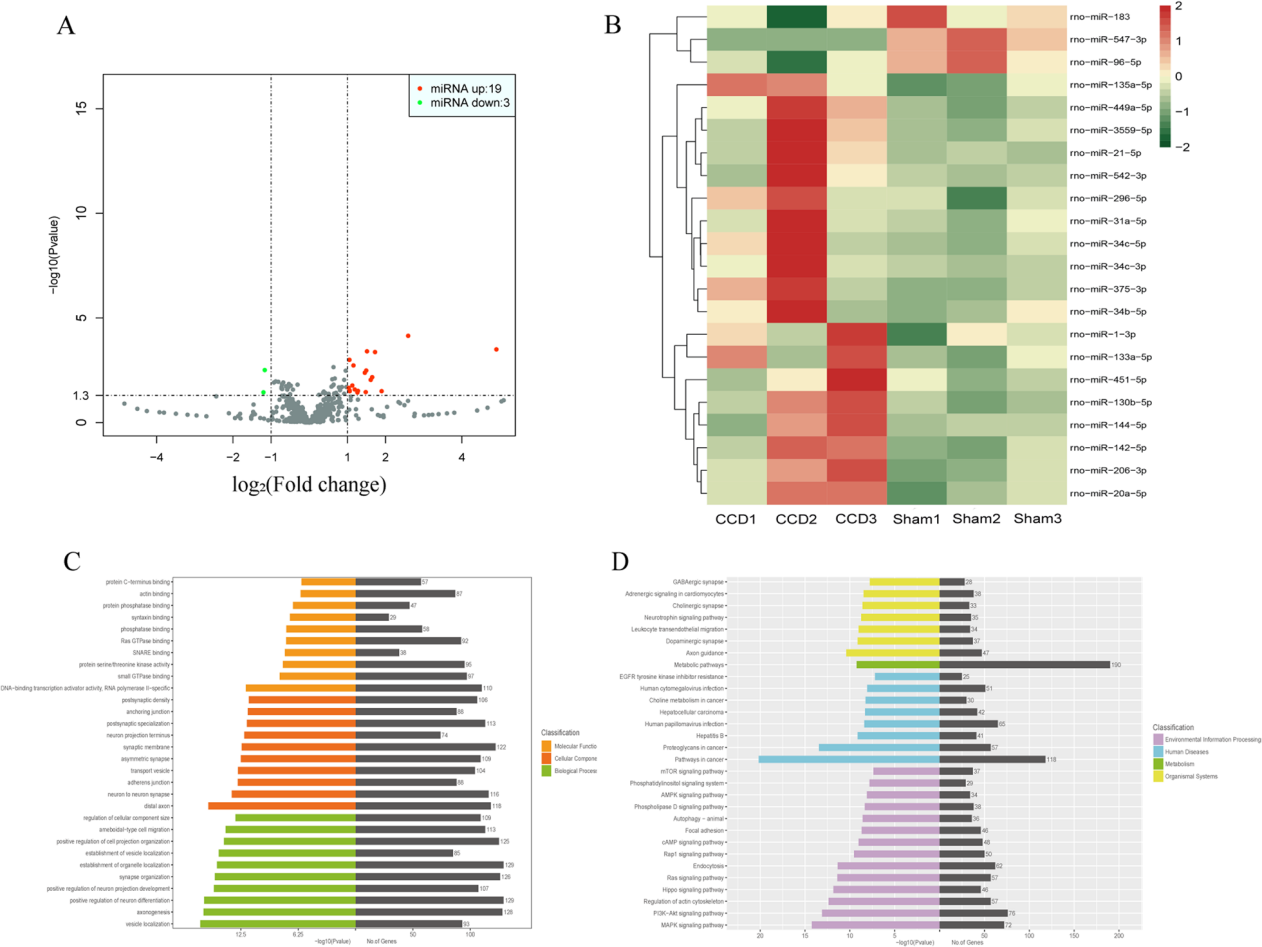
Analysis of differentially expressed miRNAs in DRG between the CCD and sham groups. **A** Volcano plot of differentially expressed miRNAs in each group. Red plots indicate upregulated miRNAs and green plots indicate downregulated miRNAs in CCD group compared with those in the sham group. **B** Heat map of differentially expressed miRNAs. For each miRNA, red indicates upregulated and green indicates downregulated miRNAs. The darker the color, the higher would be the expression. **C**, **D** GO functional enrichment and KEGG pathway analysis were performed to identify potential pathways or biological processes associated with CCD-induced neuropathic pain. The abscissa indicates the number of enriched target genes and − log_10_(*P*-value), and the ordinate indicates the top 30 GO enrichment or KEGG pathway terms. *CCD* chronic compression of dorsal root ganglion, *DRG* dorsal root ganglion, *GO* Gene Ontology, *KEGG* Kyoto Encyclopedia of Genes and Genomes

Between the CCD and sham groups, 22 differentially expressed miRNAs were identified. Compared with the sham group, 19 miRNAs were upregulated and 3 were downregulated in DRG of the CCD group (Fig. 2A, B; detailed information shown in Table 2). To speculate the molecular mechanisms of the differentially expressed miRNAs, their target genes were predicted. The number of target genes of each miRNA was finally obtained via the screening of the predicted results (detailed information is presented in Additional file 2). GO functional enrichment and KEGG pathway analysis were then performed to identify potential biological processes or pathways associated with CCD-induced neuropathic pain. Among the biological functions obtained using GO analysis (Fig. 2C), molecular functions mainly included DNA-binding transcription activator activity, small GTPase binding, and protein serine/threonine kinase activity; cellular components mainly included synaptic membrane, distal axon, and neuron-to-neuron synapse; and biological processes mainly included positive regulation of neuron differentiation, the establishment of organelle localization, and axogenesis. Besides, using KEGG pathway analysis (Fig. 2D), it was determined that the target genes of differentially expressed miRNAs mainly participated in physiological processes, such as metabolism, inflammation, and apoptosis, in DRG through several pathways in cancer, PI3K–Akt and MAPK signaling pathways, so as to play a role in peripheral nerve injury caused by CCD. The specific modes of action of the predicted target genes in the pathway are shown in Additional file 4.

**Table 2:** List of miRNAs differentially expressed in DRG of CCD rats compared with sham rats

No.	MiRNA	Log_2_ (fold change)	Regulation	*P*-value
1	miR-375-3p	4.903986176	Up	< 0.001
2	miR-449a-5p	2.592291784	Up	< 0.001
3	miR-21-5p	1.897166256	Up	0.031
4	miR-135a-5p	1.723550334	Up	< 0.001
5	miR-34c-3p	1.650507208	Up	0.007
6	miR-31a-5p	1.611819174	Up	0.009
7	miR-206-3p	1.513704055	Up	< 0.001
8	rmiR-34c-5p	1.493525558	Up	0.003
9	miR-144-5p	1.480404628	Up	0.035
10	miR-130b-5p	1.456294712	Up	0.004
11	miR-3559-5p	1.276851951	Up	0.030
12	miR-34b-5p	1.262181137	Up	0.036
13	miR-133a-5p	1.191435092	Up	0.026
14	miR-142-5p	1.158505314	Up	0.002
15	miR-1-3p	1.129191416	Up	0.017
16	miR-542-3p	1.059693765	Up	0.032
17	miR-296-5p	1.056567783	Up	0.029
18	miR-20a-5p	1.053024673	Up	0.001
19	miR-451-5p	1.035219621	Up	0.022
20	miR-96-5p	− 1.166135601	Down	0.003
21	miR-183	− 1.200157191	Down	0.036
22	miR-547-3p	− 6.675122118	Down	< 0.001

**Fig. 3:**
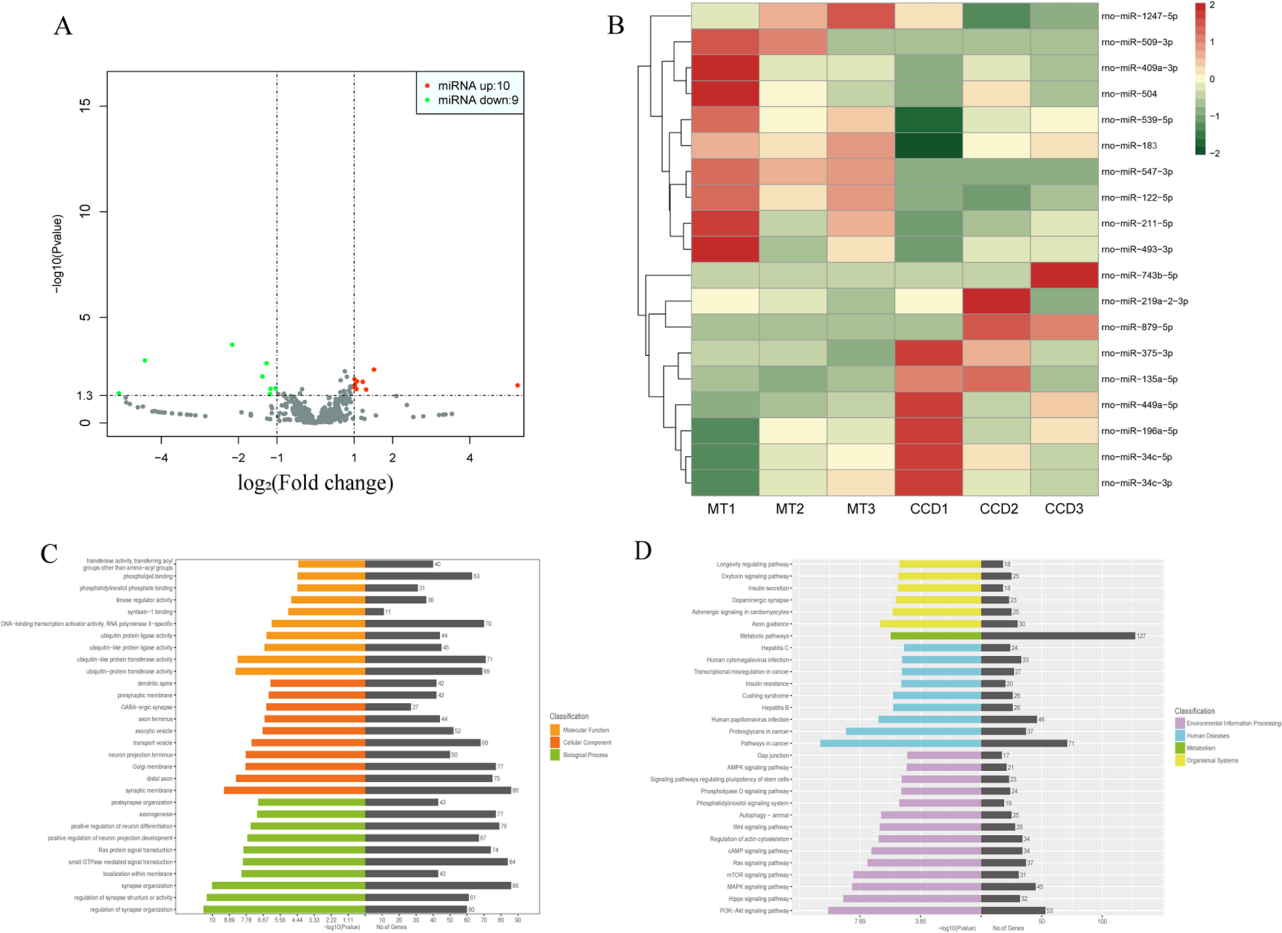
Analysis of differentially expressed miRNAs in DRG between the MT and CCD groups. **A** Volcano plot of differentially expressed miRNAs. Red plots indicate upregulated miRNAs and green plots indicate downregulated miRNAs in the MT group compared with those in the CCD group. **B** Heat map of differentially expressed miRNAs. For each miRNA, red indicates upregulated and green indicates downregulated miRNAs. The darker the color, the higher would be the expression. **C**, **D** GO functional enrichment and KEGG pathway analysis were performed to identify potential pathways or biological processes associated with the mechanism of MT. The abscissa indicates the number of enriched target genes and − log_10_(*P*-value), and the ordinate indicates the top 30 GO enrichment or KEGG pathway terms. *CCD* chronic compression of dorsal root ganglion, *DRG* dorsal root ganglion, *GO* Gene Ontology, *KEGG* Kyoto Encyclopedia of Genes and Genomes, *MT* manual therapy

Between the MT and CCD groups, 19 differentially expressed miRNAs were identified. Compared with the CCD group, 10 miRNAs were upregulated and 9 were downregulated in DRG of the MT group (Fig. 3A, B; detailed information shown in Table 3). To decipher the molecular mechanisms of the differentially expressed miRNAs, their target genes were predicted (Additional file 5). GO functional enrichment and KEGG pathway analysis were performed to identify potential biological processes or pathways involved in the MT mechanism. Among the biological functions obtained using GO analysis (Fig. 3C), molecular functions mainly included ubiquitin-like protein transferase activity, DNA-binding transcription activator activity, and ubiquitin-protein transferase activity; cellular components mainly included the synaptic membrane, Golgi membrane, and distal axon; and biological processes mainly included synapse organization, small GTPase-mediated signal transduction, and regulation of neuron differentiation. Besides, using KEGG pathway analysis (Fig. 3D), we found that the target genes also play a role in the improvement of MT on DRG injury through several pathways in cancer, PI3K–Akt and MAPK signaling pathways. In addition, compared with the changes observed in the CCD group, the enrichment degree of pathways associated with inflammation was increased, such as the mTOR and cAMP signaling pathways. The specific modes of action of the predicted target genes in the pathway are shown in Additional file 3.

**Table 3:** List of miRNAs differentially expressed in DRG of MT rats compared with CCD rats

No.	MiRNA	Log_2_(fold change)	Regulation	*P*-value
1	miR-547-3p	6.675243661	Up	< 0.001
2	miR-509-3p	5.238731719	Up	0.016
3	miR-122-5p	1.514745189	Up	0.003
4	miR-1247-5p	1.312949213	Up	0.026
5	miR-493-3p	1.223811626	Up	0.011
6	miR-539-5p	1.071325388	Up	0.011
7	miR-504	1.062272899	Up	0.025
8	miR-183	1.02056182	Up	0.021
9	miR-409a-3p	1.019991442	Up	0.016
10	miR-211-5p	1.010332199	Up	0.008
11	miR-196a-5p	− 1.036709766	Down	0.023
12	miR-219a-2-3p	− 1.169051867	Down	0.024
13	miR-34c-3p	− 1.178103005	Down	0.043
14	miR-135a-5p	− 1.270267364	Down	0.001
15	miR-34c-5p	− 1.375983005	Down	0.006
16	miR-449a-5p	− 2.155228628	Down	< 0.001
17	miR-375-3p	− 4.421941598	Down	0.001
18	miR-879-5p	− 5.09743657	Down	0.039
19	miR-743b-5p	− 5.715888915	Down	0.014

### Screening of key miRNA of MT to alleviate CCD-induced neuropathic pain

**Fig. 4:**
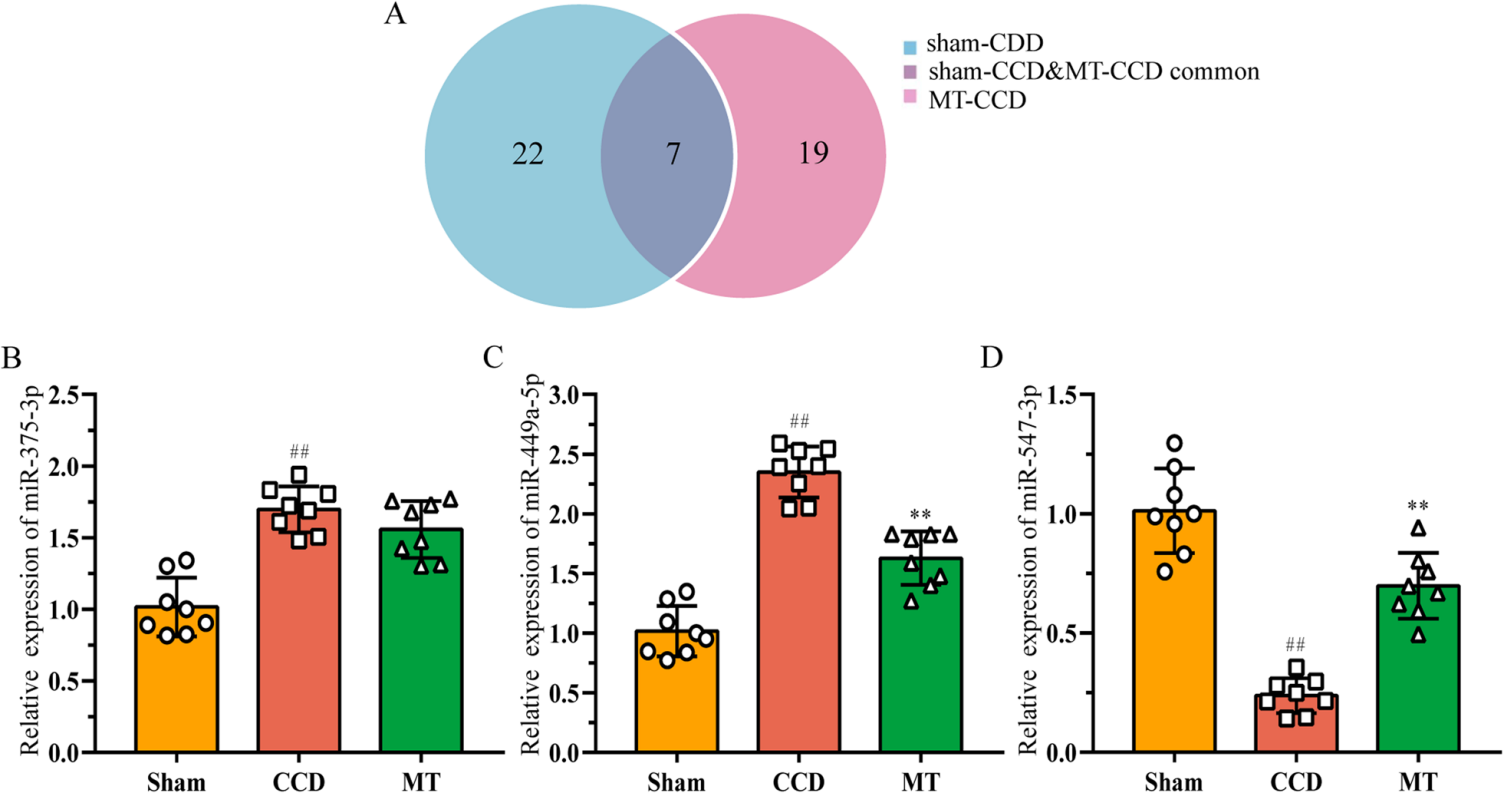
Validations of miRNAs with high fold change between different groups. **A** Venn diagram of miRNAs differentially expressed between the groups. **B**–**D** Relative expression of miR-375-3p, miR-449a-5p, and miR-547-3p was detected using qRT-PCR to validate the sequencing results. The data are expressed as mean ± SD (*N* = 8) and analyzed for statistical significance using one-way ANOVA followed by LSD post hoc analysis for multiple comparisons (^##^*P* < 0.01 versus sham group, ^**^P < 0.01 versus CCD group). *CCD* chronic compression of dorsal root ganglion, *LSD* least significant difference, *MT* manual therapy, *qRT-PCR* real-time quantitative reverse transcription PCR

To screen the key miRNA that may be associated with the alleviation of CCD-induced neuropathic pain by MT, miRNAs with high FC between diverse groups were selected. As shown in Fig. 4A, the comparison of the differentially expressed miRNAs between the sham–CCD and CCD–MT groups highlighted seven miRNAs (detailed information is shown in Table 4). Among these, miR-375-3p, miR-449a-5p, and miR-547-3p showed the highest FC. They were analyzed using qRT-PCR to validate the reliability of sequencing (Fig. 4B–D), and the results showed that the expression of miRNAs was largely consistent with the sequencing data.

**Table 4:** List of miRNAs differentially expressed in DRG between the different groups

No.	MiRNA	Sham–CCD		CCD–MT	
		Log_2_ (fold change)	Regulation	Log_2_ (fold change)	Regulation
1	miR-375-3p	4.903986176	Up	− 4.421941598	Down
2	miR-449a-5p	2.592291784	Up	− 2.155228628	Down
3	miR-135a-5p	1.723550334	Up	− 1.270267364	Down
4	miR-34c-3p	1.650507208	Up	− 1.178103005	Down
5	miR-34c-5p	1.493525558	Up	− 1.375983005	Down
6	miR-183	− 1.200157191	Down	1.02056182	Up
7	miR-547-3p	− 6.675122118	Down	6.675243661	Up

To further investigate the pathways regulated by the key miRNA in controlling the effects of MT, we focused more closely on the prediction of the target genes of miR-375-3p, miR-449a-5p, and miR-547-3p (Additional file 6) and analyzed their KEGG pathways. The largest number of predicted target genes was identified for miR-547-3p (Table 5). Furthermore, qRT-PCR analysis showed that the expression of miR-547-3p significantly decreased in DRG of the CCD group (*P* < 0.01) and increased after MT intervention (*P* < 0.01; Fig. 4D). Therefore, we speculated that miR-547-3p is a key miRNA in mediating analgesia and anti-inflammatory response of MT to neuropathic pain.

**Table 5:** Prediction of key miRNAs target genes and pathways involved

MiRNA	No. of genes	Target genes	KEGG pathways
miR-375-3p	86	Atpaf1, Cacnb4, Cstf2, Foxe1, Fzd8, Golph3, Otud7b, Pax6, Ube3a, Ywhaz	mRNA surveillance pathway
miR-449a-5p	45	Fam45a, Gmppb, Hdac1, Lrrc59, Lrtm2, Ltbp2, Notch1, Pde1c, Plcb1, Plod1	Thyroid hormone signaling pathway Adrenergic signaling in cardiomyocytes
miR-547-3p	172	Adcy2, Ap1s3, Cdh2, Igf1、Kitlg, Map4k4, Pdgfrb、Rptor, Smad2, Ube2h	Pathways in cancer PI3K–Akt signaling pathway Rap1 signaling pathway TGF-beta signaling pathway Ubiquitin-mediated proteolysis Signaling pathways regulating, pluripotency of stem cell Central carbon metabolism in cancer

Due to the large number of target genes, only 10 are listed for each miRNA in the table

### Validation of Map4k4 as a potential neuroinflammation and pain-related target gene of miR-547-3p

**Fig. 5:**
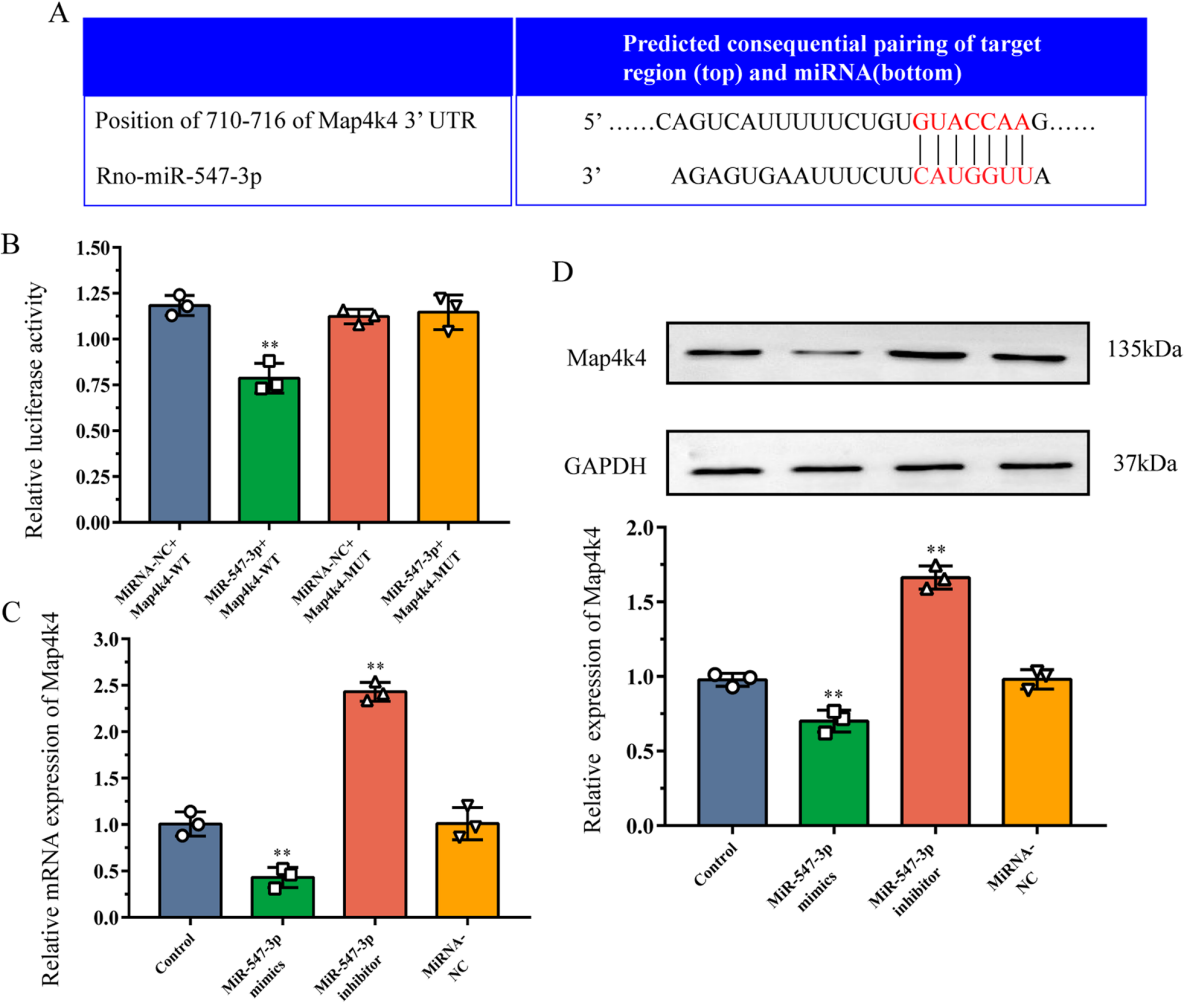
Validation of Map4k4 as a target gene of miR-547-3p. **A** Prediction of binding sites between miR-547-3p and Map4k4. **B** Luciferase reporter gene assay was performed to validate the interaction between miR-547-3p and Map4k4. The data are expressed as mean ± SD (*N* = 3) and analyzed for statistical significance using one-way ANOVA followed by LSD post hoc analysis for multiple comparisons (^**^P < 0.01 versus miRNA-NC + Map4k4-WT group). **C**, **D** Western blot and qRT-PCR analysis show the effects of miR-547-3p mimics or inhibitor on the expression of Map4k4 in the DRG cells. The data are expressed as mean ± SD (*N* = 3) and analyzed for statistical significance using one-way ANOVA followed by LSD post hoc analysis for multiple comparisons (^**^P < 0.01 versus control group). *DRG* dorsal root ganglion, *LSD* least significant difference, *NC* negative control, *WT* wild-type, *MUT* mutant, *qRT-PCR* real-time quantitative reverse transcription PCR, *SD* standard deviation

Map4k4, one of the target genes of miR-547-3p, belongs to the mammalian serine/threonine kinase family [36]. It plays an important role in regulating the downstream MAPK and NF-κB signaling pathways [36] and can thus be used as a potential target gene for studying neuroinflammation and pain. As the vast majority of the identified miRNAs regulate the function of mRNAs through interaction with canonical sites, the binding sites between miR-547-3p and Map4k4 were predicted using TargetScan. This showed that amino acids 710–716 of Map4k4 3′UTR could bind miR-547-3p (Fig. 5A). Therefore, we decided to further verify whether miR-547-3p can effectively regulate Map4k4 directly via in vitro experiments.

For this purpose, we cotransfected a plasmid coding for WT Map4k4 and miR-547-3p mimics in HEK293T cells and observed that the relative luciferase activity was significantly reduced (*P* < 0.01; Fig. 5B). Therefore, the specific decrease in luciferase activity detected when miR-547-3p was coexpressed with Map4k4 WT suggests that miR-547-3p can specifically bind to the 3′UTR region of Map4k4, thereby inhibiting its mRNA translation. To confirm the regulatory role of miR-547-3p in Map4k4 expression level in vitro, miR-547-3p was overexpressed or inhibited in DRG neurons, and the relative expression of Map4k4 was detected using qRT-PCR and Western blot (Fig. 5C–E). The relative mRNA and protein expression of Map4k4 in DRG neurons overexpressing miR-547-3p was significantly decreased compared with the control group (*P* < 0.01), whereas the level of Map4k4 increased significantly (*P* < 0.01) in those transfected with miR-547-3p inhibitor. In addition, the variation in the relative expression of Map4k4 in the negative control group was not statistically significant compared with the control (*P* > 0.05). Altogether, these results suggest that miR-547-3p can directly inhibit the expression of Map4k4 in DRG cells; thus, it is involved in the regulation of neuroinflammation and pain.

### MT reduces neuropathic pain by regulating miR-547-3p targeting Map4k4

**Fig. 6:**
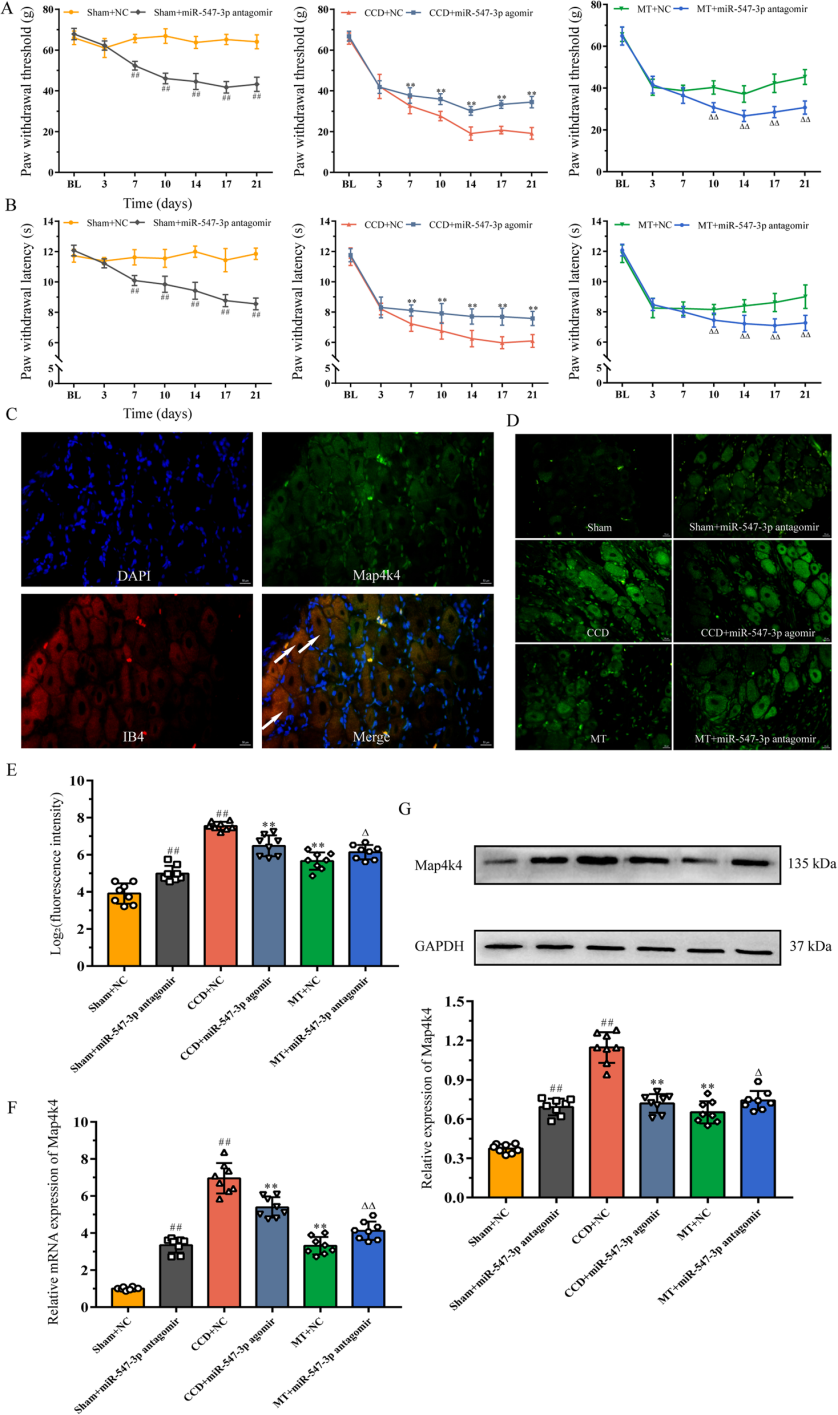
MT reduces neuropathic pain by regulating miR-547-3p targeting Map4k4. **A**, **B** PWT and PWL of rats were measured to assess their mechanical and thermal hyperalgesia. The data are expressed as mean ± SD (*N* = 8). Repeated measurement ANOVA was used to analyze the data at various time points between the groups and one-way ANOVA followed by LSD post hoc analysis was used for pairwise comparison (at the same time point, ^##^*P* < 0.01 versus sham + NC group, ^**^P < 0.01 versus CCD + NC group, and ^ΔΔ^*P* < 0.01 versus MT + NC group). **C**, **D** Immunofluorescence staining reveals the expression and cellular distribution of Map4k4 in different groups; white arrows indicate the co-expression of Map4k4 (green fluorescent) and IB4-positive nociceptors (red fluorescent) in DRG neurons (scale bar = 50 μm). **E** Quantification analysis of Map4k4 fluorescence intensity. The data are expressed as mean ± SD (*N* = 8) and analyzed for statistical significance using one-way ANOVA followed by Dunnett’s T3 post hoc analysis for multiple comparisons (^##^*P* < 0.01 versus sham + NC group, ^**^P < 0.01 versus CCD + NC group, ^Δ^*P* < 0.05 versus MT + NC group). **F**,** G** qRT-PCR and Western blot analysis show the relative mRNA and protein expression of Map4k4, respectively. The data are expressed as mean ± SD (*N* = 8). One-way ANOVA followed by LSD post hoc analysis was used for protein expression, and one-way ANOVA followed by Dunnett’s T3 post hoc analysis was used for relative mRNA expression (^##^*P* < 0.01 versus sham + NC group, ^**^P < 0.01 versus CCD + NC group, ^Δ^*P* < 0.05, and ^ΔΔ^*P* < 0.01 versus MT + NC group). *ANOVA* analysis of variance, *CCD* chronic compression of dorsal root ganglion, *LSD* least significant difference, *MT* manual therapy, *NC* negative control, *SD* standard deviation

To evaluate the effects of variations in the levels of miR-547-3p on neuropathic pain, rats were intrathecally injected with miR-547-3p agomir or antagomir, and their behavior was tested (Fig. 6A, B). PWT and PWL of the sham group intrathecally injected with miR-547-3p antagomir decreased until day 14 and were significantly lower than those of the sham group injected with antagomir control at the same time point from day 7. PWT and PWL of the CCD group showed a downward trend after intrathecal injection of miR-547-3p agomir, but they were significantly higher than the CCD group injected with agomir control at the same time point from day 7. Besides, PWT and PWL of rats in the MT group injected intrathecally with miR-547-3p antagomir were significantly lower than those injected with agomir control from day 10.

Next, to validate the effects of miR-547-3p regulation on its target gene Map4k4 in rat DRG, mRNA expression of Map4k4 was measured using qRT-PCR and protein expression was measured using immunofluorescence staining and Western blot. Immunofluorescence staining showed abundant co-expression of Map4k4 with IB4-positive nociceptors involved in pain signaling in DRG neurons, suggesting that Map4k4 is mainly distributed in IB4-positive neurons (Fig. 6C). Quantitative analysis of fluorescence intensity showed that intrathecal injection of miR-547-3p agomir as well as MT intervention resulted in a significant reduction of Map4k4 expression in the CCD group (*P* < 0.01), whereas Map4k4 expression significantly increased in rats intrathecally injected with miR-547-3p antagomir in the sham and MT groups (*P* < 0.05, *P* < 0.01; Fig. 6D, E). In addition, the detection of relative protein and mRNA expression of Map4k4 using Western blot and qRT-PCR revealed that both miR-547-3p overexpression and MT intervention could significantly inhibit Map4k4 protein and mRNA expression in rat DRG (*P* < 0.01), and the inhibition of miR-547-3p with antagomir significantly increased protein and mRNA expression of Map4k4 in the sham and MT groups (*P* < 0.05, *P* < 0.01; Fig. 6F, G).

These results suggest that the inhibition of miR-547-3p or MT intervention can alleviate mechanical and thermal pain in the CCD group. In addition, blocking miR-547-3p in the sham group can induce pain sensitivity and inhibit the improvement in pain induced by MT. The above processes could be completed through the regulation of Map4k4 by miR-547-3p. Therefore, we speculated that the analgesic effect of MT could be exerted by inhibiting Map4k4 expression through the upregulation of miR-547-3p.

### Inhibition of Map4k4/NF-κB signaling pathway may be a potential mechanism of miR-547-3p-mediated anti-inflammatory effect of MT

As described above, by analyzing the possible mechanisms of miR-547-3p, we speculated that it could participate in the development and maintenance of neuropathic pain. In this process, it would affect the DRG local inflammatory microenvironment through the regulation of inflammation-related NF-κB signaling pathway by targeting Map4k4. To verify this hypothesis, we focused on two core factors of the NF-κB pathway, p65 and IκBα, and examined their expression and phosphorylation upon activation as well as the content of downstream inflammatory cytokines in rat DRG.

**Fig. 7:**
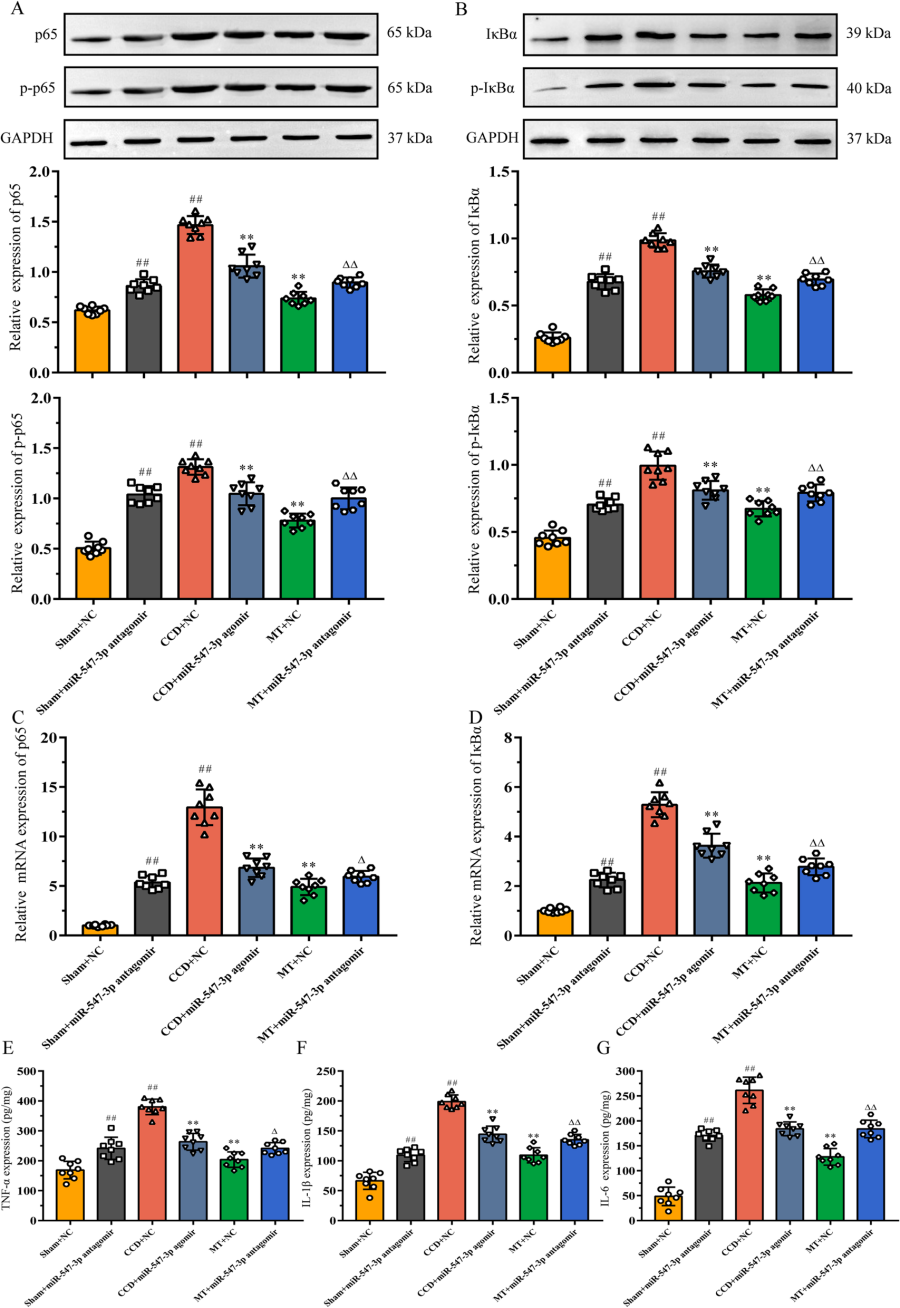
Inhibition of Map4k4/NF-κB signaling pathway may be a potential mechanism of miR-547-3p-mediated anti-inflammatory effect of MT. **A**, **B** Western blot analysis showed the relative expression of p65, p-p65, IκBα, and p-IκBα. The data are expressed as mean ± SD (*N* = 8) and analyzed for statistical significance using one-way ANOVA followed by LSD post hoc analysis for multiple comparisons (^##^*P* < 0.01 versus sham + NC group, ^**^P < 0.01 versus CCD + NC group, ^ΔΔ^*P* < 0.01 versus MT + NC group). **C**, **D** qRT-PCR results showed the relative mRNA expression of p65 and IκBα. The data are expressed as mean ± SD (*N* = 8) and analyzed for statistical significance using one-way ANOVA followed by Dunnett’s T3 post hoc analysis for multiple comparisons (^##^*P* < 0.01 versus sham + NC group, ^**^P < 0.01 versus CCD + NC group, ^Δ^*P* < 0.05 and ^ΔΔ^*P* < 0.01 versus MT + NC group). **(E–G)** ELISA revealed the expression of TNF-α, IL-1β, and IL-6 in DRG. The data are expressed as mean ± SD (*N* = 8). One-way ANOVA followed by LSD post hoc analysis was used for TNF-α and IL-1β, and one-way ANOVA followed by Dunnett’s T3 post hoc analysis was used for IL-6 (^##^*P* < 0.01 versus sham + NC group, ^**^P < 0.01 versus CCD + NC group, ^Δ^*P* < 0.05, and ^ΔΔ^*P* < 0.01 versus MT + NC group). *ANOVA* analysis of variance, *CCD* chronic compression of dorsal root ganglion, *DRG* dorsal root ganglion, *ELISA*: enzyme-linked immunosorbent assay, *LSD* least significant difference, *MT* manual therapy, *NC* negative control

Using Western blot, the protein expression of p65, p-p65, IκBα, and p-IκBα was detected in the DRG tissues of rats in each group (Fig. 7A, B). Compared with the sham group, the expression of p65, p-p65, IκBα, and p-IκBα in the CCD and sham groups injected with miR-547-3p antagomir increased significantly (*P* < 0.01). In contrast, compared with the CCD group, injection of miR-547-3p agomir and MT intervention significantly decreased the expression of p65, p-p65, IκBα, and p-IκBα (*P* < 0.01). Besides, the injection of miR-547-3p antagomir could inhibit the regulatory effect of MT on the abovementioned proteins (*P* < 0.01). Furthermore, qRT-PCR analyses showed that the mRNA expression of p65 and IκBα follows similar expression trends as proteins (*P* < 0.05, *P* < 0.01; Fig. 7C, D). Finally, the expression of inflammatory cytokines, such as TNF-α, IL-1β, and IL-6, in DRG was detected using ELISA (Fig. 7E–G). The level of inflammatory cytokines increased significantly with the activation of the NF-κB pathway (*P* < 0.05, *P* < 0.01), and inhibiting the activation of this pathway significantly decreased the level of inflammatory cytokines (*P* < 0.01).

These results indicate that MT could exert an effect similar to that of mir-547-3p agonist, regulate targeted Map4k4 through miR-547-3p, and inhibit the activation of the NF-κB pathway, thus playing anti-inflammatory and analgesic roles.

## Discussion

In this study, we first evaluated the behavioral changes in a rat model of neuropathic pain induced by CCD. We showed that the inflammatory cytokine levels in DRG correlates with pain behavior in rats and confirmed that MT may alleviate pain by improving neuroinflammation. To investigate the potential mechanisms of MT, we applied high-throughput sequencing technology and discovered that MT exert its effects through miRNAs, and further screening key miRNAs mediating anti-inflammatory and analgesic effects of MT. We then performed in vitro cell experiments to validate that miR-547-3p could regulate Map4k4 in DRG cells. Finally, we overexpressed or antagonized miR-547-3p in rats and examined the expression and phosphorylation of core factors of the Map4k4/NF-kB pathway, confirming that this may be one of the mechanisms by which MT exerts its anti-inflammatory and analgesic effects.

CCD is a classic model to study neuropathic pain [22]. By inserting stainless-steel rods into the corresponding intervertebral foramen of rats, it can create persistent physical compression on DRG, leading to local edema of the nerve and secondary inflammatory reaction, thus causing radiating pain in the lower back and legs. Different from other models [37–39], CCD has the advantage of creating a local inflammatory microenvironment by directly compressing nerve roots and yet preserving the transmission function of peripheral nerves. This closely resembles the clinical symptoms and signs of neuropathic pain caused by LDH [40]. In this study, the thresholds of mechanical and thermal pain of the rats continued to decrease until they reached a relatively stable state after 14 days after CCD surgery, which is consistent with the results obtained in previous studies [41, 42]. In addition, ELISA experiments showed that the expression of inflammatory cytokines, such as TNF-α, IL-1β, and IL-6, in DRG of the CCD group were significantly higher than those of the sham group. Therefore, it can be speculated that the chronic compression of DRG can form aseptic inflammation to elevate the levels of local proinflammatory factors, thereby inducing neuropathic pain.

MT is an important part of traditional medicine in several countries, and it has been widely applied in China for thousands of years because of its efficacy in clinical treatments, particularly for pain relief [43, 44]. Similar to acupuncture [45, 46], MT in China is mainly based on the theory of meridians and acupoints of traditional Chinese medicine, which plays its role on various reaction points of the body surface. However, unlike acupuncture, even at the same point, MT can be implemented in several ways, notably by pressing, pinching, and kneading; thus, it is better known as Tuina (pressing and holding) in China. In this situation, the quantification and standardization of MT are the challenges for current research. Recently, we have conducted research in the field of MT intervention in pain [25–27] and confirmed its exact efficacy and few possible mechanisms, but further research is needed. In this study, “Weizhong” (BL40) was selected as the implementation site of MT because of its specific efficacy in low back and leg pain according to previous studies [47–49] and because BL40 is located behind the knee joint, which can avoid affecting wound healing after surgery. Our results confirmed that by performing MT at BL40, the pain behavior as well as the levels of inflammatory factors of the CCD group could be reduced. As MT did not change the compression status of nerve roots provoked by the steel rods, improving the local inflammatory microenvironment of DRG might be the critical mechanism by which MT exerts an analgesic effect. Moreover, this demonstrates that the secondary inflammatory response may be more important in LDH-induced neuropathic pain than the nerve root compression itself.

Recently, studies on the involvement of miRNAs in pain-related diseases have been rapidly increasing [34, 50, 51]. Currently, they are mainly focused on investigating the roles of individual miRNA in various physiological and pathological conditions as well as on determining whether they could be potential therapeutic targets. Besides, miRNAs can also serve as pain diagnostic tools to assess pain states, identify chronic pain, and guide the use of drugs. As the primary center of sensory conduction, DRG neurons contain numerous neurotransmitters and ion channel proteins, which are responsible for the generation and transmission of pain signals; thus, they play an important role in the study of neuropathic pain. In our study, 22 differentially expressed miRNAs have been identified in DRG of the CCD group compared with the sham group. Among these, the upregulation of miR-21-5p [52, 53] and miR-31a-5p [54] and the downregulation of miR-96-5p were consistent with the results of previous research obtained in other models. This not only confirms the credibility of the CCD model, but also indicates the similarity in the roles of these miRNAs in neuropathic pain. However, miR-375-3p, miR-449a-5p, and miR-547-3p with the highest FCs were not frequently reported in neuropathic pain in previous studies [35, 55, 56], which may be due to the differences in model type and sample size. Next, the differential expression of these miRNAs in DRG of the CCD group was confirmed using qRT-PCR. Further, GO enrichment analysis of their target genes showed enrichment in neuron-related biological functions, which was in accordance with the damage of peripheral nerves caused by CCD. Moreover, KEGG pathway analysis demonstrated that these genes may contribute to the pathology of neuropathic pain, mainly via pathways in cancer, PI3K–Akt and MAPK signaling pathways.

Based on the above results, we aimed to explore the potential mechanisms of MT on the alleviation of inflammation and pain from the perspective of miRNAs. In this study, 19 differentially expressed miRNAs were identified in the DRG of rats between the MT and CCD groups. Similarly, we deciphered the function of these target genes through GO and KEGG pathway analysis and found that their enrichment in functions such as ubiquitin-protein transferase activity was special, which may involve the regulation of NF-κB and downstream proinflammatory cytokines [57]. Notably, seven miRNAs were differentially expressed in different groups, among which miR-375-3p, miR-449a-5p, and miR-547-3p showed the most obvious FCs in the groups. qRT-PCR revealed that the expression of miR-449a-5p and miR-547-3p in DRG of the CCD group after MT intervention was significantly increased; however, the downregulation of miR-375-3p expression was not statistically significant. The sample size may need to be further increased in the future studies. KEGG analysis was performed again for the target genes of each miRNA, and we speculated that miR-547-3p is the key miRNA mediating MT to improve the inflammatory microenvironment induced by CCD and alleviate pain in DRG. In our study, MT could exert anti-inflammatory and analgesic effects by upregulating the expression of miR-547-3p to inhibit the expression of TNF-α, IL-1β, IL-6; however, the pathway underlying this process remains unclear. Next, miR-547-3p target gene Map4k4 was found via bioinformatics analysis, and the direct regulation of Map4k4 by miR-547-3p was verified by luciferase reporter gene analysis. It was further confirmed in rat DRG cells that the overexpression or inhibition of miR-547-3p would affect the protein and mRNA expression of Map4k4, suggesting that the effects of MT are exerted by targeting Map4k4 by miR-547-3p. Map4k4 belongs to the mammalian serine/threonine kinase family and is involved in cell proliferation, glucose transport, inflammatory response, and other processes [58], but its specific mechanisms have not yet been investigated. Some researchers [59] believe that the proinflammatory effect of Map4k4 can be achieved by upregulating the expression of NF-κB, and silencing or inhibiting Map4k4 can reduce the expression of downstream TNF-α. Although there are few reports on the involvement of Map4k4 in neuropathic pain, it can affect the growth of neuronal axons and dendrites in the SH-SY5Y cell line derived from human neuroblastoma [60]. Notably, more than 4959 proteins have been identified in human and rat DRG through proteomic studies [61], with similarities between species. Map4k4 is unique among these proteins; its expression and functional patterns show obvious cross-species characteristics, and its inhibition can promote the growth of DRG neurons. Thus, miR-547-3p may affect the NF-κB pathway by targeted regulation of Map4k4, thereby mediating the effects of MT.

As the core transcription factor in NF-κB signaling pathway, NF-κB exists in the cytoplasm as an inactive form of trimer with its subunits p65, p50, and inhibitor of NF-κB (IκB) in the resting state. On stimulation, IκB kinase phosphorylates and ubiquitinates IκBα, releasing NF-κB into the nucleus; among the subunits, p65 is the most predominant active form [62]. The activation of NF-κB pathway initiates the transcription of its downstream proinflammatory factors, such as TNF-α, IL-1β, and IL-6, inducing inflammatory response and pain. Moreover, these inflammatory cytokines become activators of NF-κB, further activating NF-κB pathway and aggravating inflammation [63–65]. In this study, the expression of Map4k4 in DRG of the CCD group was significantly increased, resulting in the increase in the expression and phosphorylation of downstream IκBα. This process could release and phosphorylate p65, sufficiently activating the NF-κB pathway to initiate transcription of TNF-α, IL-1β, and IL-6. Through MT intervention, Map4k4 expression could be significantly reduced, resulting in the inhibition of NF-κB pathway-associated Iκbα, p-Iκba, p65, and p-p65 expression. To verify whether the above processes were mediated by miR-547-3p, we further overexpressed or antagonized miR-547-3p in the tissues (Additional file 5, Additional file 6).

MiRNA agomir and antagomir are chemically modified miRNA agonists and antagonists, respectively, which can be used in vivo to regulate the expression of target gene mRNA. Compared with the common miRNA mimics and inhibitor, miRNA agomir and antagomir have higher stability and miRNA activity in animals and are more easily enriched in target cells through the cell membranes and tissue gap. They can be administered in animals via systemic or local injection, and the effect can last for several weeks. The results confirmed that after intrathecal injection of miR-547-3p antagomir in rats of the sham group, the inhibition of miR-547-3p on Map4k4 was reduced, resulting in a proinflammatory effect similar to that of the CCD group, thus leading to pain behavior in rats. Besides, after injecting miR-547-3p agomir in the CCD group, Map4k4 expression decreased because of the overexpression of miR-547-3p, which produced an anti-inflammatory effect and alleviated the pain. Moreover, the injection of miR-547-3p antagomir inhibited the effects of MT to a certain extent. However, the antagonism of miR-547-3p did not completely inhibit the anti-inflammatory and analgesic effect of MT, indicating that the effect of miR-547-3p-mediated Map4k4/NF-kb signaling pathway is only one of the potential mechanisms of MT. The multichannel and multitarget intervention characteristics of MT are worthy of further exploration to provide a better theoretical basis for clinical promotion.

Notably, to the best of our knowledge, this is the first study to use high-throughput sequencing technology to explore miRNAs and their mechanisms that mediate the analgesic effect of MT, so that only the classic effect of anti-inflammatory response is selected. Other mechanisms of MT can be further explored according to the current research results. Moreover, although there is literature supporting the regulation of Map4k4 in the NF-κB pathway, it has not yet been verified whether such an effect in rat DRG is caused by other target genes mediated by miR-547-3p, which could be assessed using Map4k4 knockout rats in the future studies.

## Conclusions

MiRNAs are differentially expressed in DRG of CCD-induced neuropathic pain rats, and MT can intervene the transcription and translation of inflammation-related genes at the epigenetic level through miRNAs to improve neuroinflammation and alleviate neuropathic pain. MiR-547-3p is a key target of MT for anti-inflammatory and analgesic effects, and the mechanism underlying this process could be achieved by mediating Map4k4/NF-κB pathway involvement in regulating downstream inflammatory cytokines. Our findings reveal the mechanisms underlying CCD-induced neuropathic pain and confirm the uniqueness of non-drug therapy for the management of pain-related diseases in the future.

## Supplementary Information

**Additional file 1.** Observation and verification of dorsal root ganglion neurons.

**Additional file 2.** The sequences of miRNA mimics, miRNA inhibitor, scrambled miRNA, Map4k4 wild-type and mutant.

**Additional file 3.** Quality inspection results of the samples.

**Additional file 4.** The the number of predicted target genes of differentially expressed miRNAs (CCD-sham).

**Additional file 5.** The the number of predicted target genes of differentially expressed miRNAs (MT-CCD).

**Additional file 6.** The prediction of the target genes of miR-375-3p, miR-449a-5p, and miR-547-3p.

## Data Availability

The main data are contained in the figures, tables, and Additional files. More details can be further obtained from the corresponding authors on request.

## Abbreviations

ANOVA: Analysis of variance
CCD: Chronic compression of dorsal root ganglia
DRG: Dorsal root ganglia
ELISA: Enzyme-linked immunosorbent assay
FC: Fold change
GO: Gene Ontology
HEK: Human embryonic kidney
IL: Interleukin
IκB: Inhibitor of NF-κB
KEGG: Kyoto Encyclopedia of Genes and Genomes
LDH: Lumbar disk herniation
LSD: Least significant difference
MiRNA: MicroRNA
MT: Manual therapy
NC: Negative control
NF-κB: Nuclear factor kappa-B
PWL: Paw withdrawal latency
PWT: Paw withdrawal threshold
SD: Standard deviation
TNF: Tumor necrosis factor
UTR: Untranslated region
WT: Wild-type
