# Supplementary figures and images for: Transcriptome profiling of microRNAs reveals potential mechanisms of manual therapy alleviating neuropathic pain through microRNA-547-3p-mediated Map4k4/NF-κb signaling pathway
Source: J Neuroinflammation. 2022 Sep 1;19:211. doi: 10.1186/s12974-022-02568-x (PMC9434879; doi:10.1186/s12974-022-02568-x)

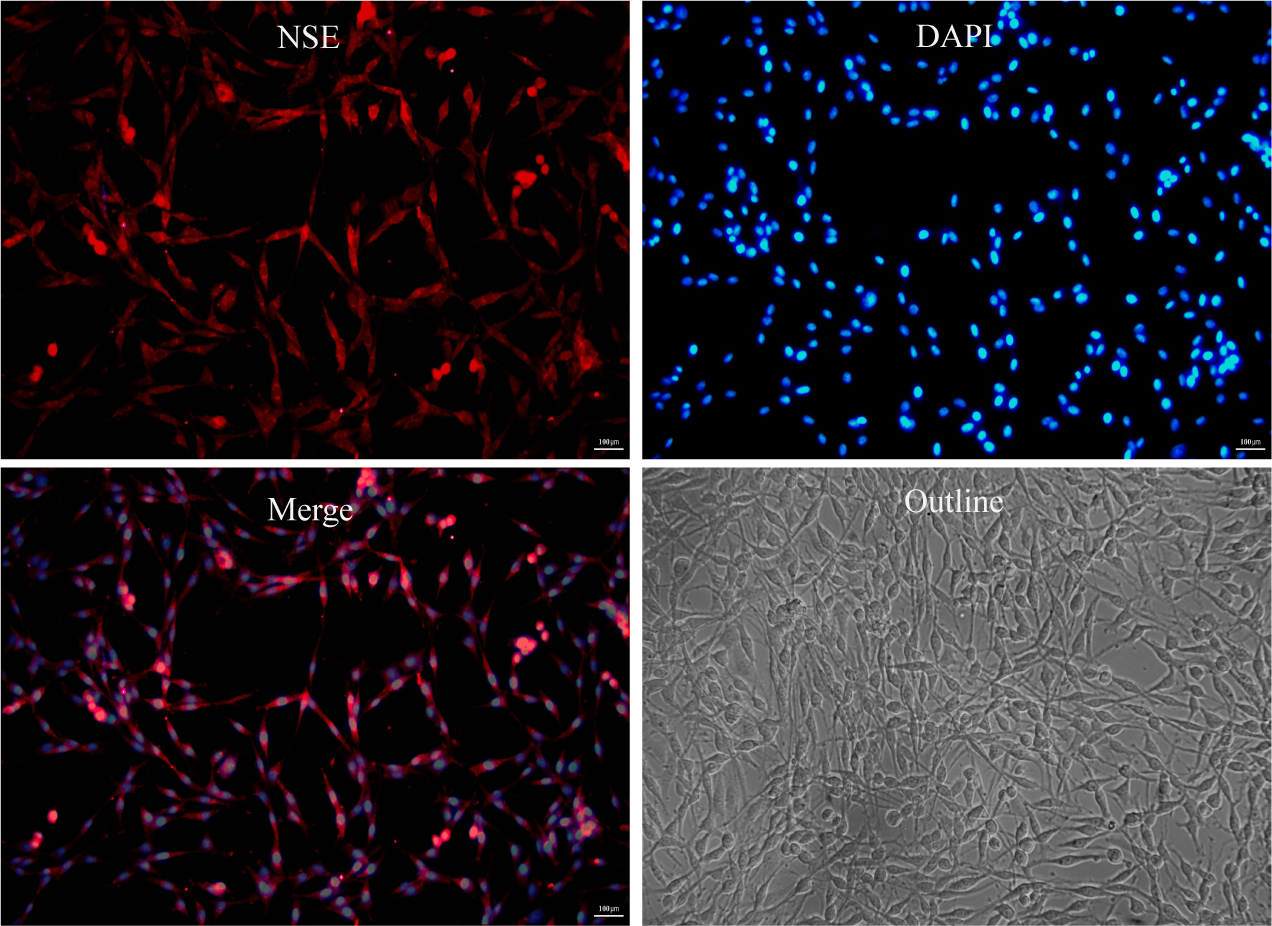

Supplement: Supplementary file 1 — Additional file 1. Observation and verification of dorsal root ganglion neurons. [file 12974_2022_2568_MOESM1_ESM.tif]
