# Supplementary material for: Transcriptome profiling of microRNAs reveals potential mechanisms of manual therapy alleviating neuropathic pain through microRNA-547-3p-mediated Map4k4/NF-κb signaling pathway
Source: J Neuroinflammation. 2022 Sep 1;19:211. doi: 10.1186/s12974-022-02568-x (PMC9434879; doi:10.1186/s12974-022-02568-x)
Supplement: Supplementary file 2 — Additional file 2. The sequences of miRNA mimics, miRNA inhibitor, scrambled miRNA, Map4k4 wild-type and mutant. [file 12974_2022_2568_MOESM2_ESM.docx]

**The sequences of miRNA mimics, miRNA inhibitor, scrambled miRNA,**

**Map4k4 wild-type and mutant**

| Gene name | Primer sequence (5′–3′) |
| --- | --- |
| miR-547-3p mimics | AUUGGUACUUCUUUAAGUGAGA |
| miR-547-3p inhibitor | UCUCACUUAAAGAAGUACCAAU |
| scrambled miRNA | AAUAGUGCCGUAUAGCACCGGU |
| Map4k4-wt | AAAGGCCAGCTTTCCTGTGGGTTCTCATTTCGTTTCGTTTTGTTTTTTGCCTACCTCATTGTTCTTAATGCATTGAGAGGTGACTTAGCTTAATGGTTTTGGCAGAAAACATTTAATGTTTAATTTAACCTTAAgtaccaaGCTATCTGATTAAAGTTTGACTGACTTTGTCACAAGTCTAATCAGGTACAAAAAAAAAAGGAAAAGAAAAGAGAAAGAAAGAAAGAAAGCTGTCTATGATGGTGGGAGCAGCCTCAGCACTCAGACGCTCTGCTC |
| Map4k4-mut | AAAGGCCAGCTTTCCTGTGGGTTCTCATTTCGTTTCGTTTTGTTTTTTGCCTACCTCATTGTTCTTAATGCATTGAGAGGTGACTTAGCTTAATGGTTTTGGCAGAAAACATTTAATGTTTAATTTAACCTTAAcgttcggACTATCTGATTAAAGTTTGACTGACTTTGTCACAAGTCTAATCAGGTACAAAAAAAAAAGGAAAAGAAAAGAGAAAGAAAGAAAGAAAGCTGTCTATGATGGTGGGAGCAGCCTCAGCACTCAGACGCTCTGCTC |
