# Supplementary material for: Transcriptome profiling of microRNAs reveals potential mechanisms of manual therapy alleviating neuropathic pain through microRNA-547-3p-mediated Map4k4/NF-κb signaling pathway
Source: J Neuroinflammation. 2022 Sep 1;19:211. doi: 10.1186/s12974-022-02568-x (PMC9434879; doi:10.1186/s12974-022-02568-x)
Supplement: Supplementary file 3 — Additional file 3. Quality inspection results of the samples. [file 12974_2022_2568_MOESM3_ESM.docx]

**Quality inspection of sequencing samples**

| Samples | Nano Drop | | | AGE | Agilent 2200 | | Conclusion |
| --- | --- | --- | --- | --- | --- | --- | --- |
|  | OD260/280 | OD260/230 | Total (μg) |  | RIN | 28S/18S |  |
| Sham1 | 1.71 | 0.27 | 3.71 | pass | 8.5 | 1.7 | A |
| Sham2 | 1.84 | 2 | 2.95 | pass | 7.9 | 1.7 | A |
| Sham3 | 1.78 | 0.44 | 5.08 | pass | 7.9 | 1.7 | A |
| CCD1 | 1.55 | 0.48 | 6.5 | pass | 8.4 | 1.9 | A |
| CCD2 | 1.11 | 0.52 | 7.84 | pass | 8.6 | 1.5 | A |
| CCD3 | 1.67 | 0.58 | 4.43 | pass | 8.3 | 1.4 | A |
| MT1 | 1.87 | 1.83 | 1.99 | pass | 7.4 | 1.5 | A |
| MT2 | 1.85 | 0.58 | 4.91 | pass | 8.7 | 1.6 | A |
| MT3 | 1.84 | 1.38 | 3.14 | pass | 8.9 | 1.5 | A |

**Raw reads quality statistics**

| Samples | Total reads | Total bases(bp) | Length | | GC% | Q20(%) | Q30(%) |
| --- | --- | --- | --- | --- | --- | --- | --- |
| Sham1 | 13,538,921 | 676,946,050 | 50 | 51.07 | | 98.45 | 96.05 |
| Sham2 | 11,154,344 | 557,717,200 | 50 | 51.3 | | 98.39 | 95.96 |
| Sham3 | 11,437,774 | 571,888.70 | 50 | 51.07 | | 98.38 | 96.01 |
| CCD1 | 12,146,960 | 607,348,000 | 50 | 50.8 | | 98.42 | 96.05 |
| CCD2 | 10,540,961 | 527,034,550 | 50 | 52.09 | | 98.39 | 96.15 |
| CCD3 | 10,826,048 | 541,302,400 | 50 | 51.02 | | 98.38 | 96 |
| MT1 | 10,863,358 | 543,167,900 | 50 | 50.96 | | 98.34 | 95.83 |
| MT2 | 12,482,706 | 624,135,300 | 50 | 50.73 | | 98.43 | 96.01 |
| MT3 | 11,527,072 | 576,353,600 | 50 | 51.05 | | 98.43 | 96.03 |

**Clean reads information statistics**

| Samples | Raw reads | Raw bases(bp) | Clean reads | Clean rate(%) |
| --- | --- | --- | --- | --- |
| Sham1 | 13,538,921 | 676,946,050 | 13,010,496 | 96.1 |
| Sham2 | 11,154,344 | 557,717,200 | 10,789,704 | 96.73 |
| Sham3 | 11,437,774 | 571,888.70 | 10,868,166 | 95.02 |
| CCD1 | 12,146,960 | 607,348,000 | 11,677,543 | 96.14 |
| CCD2 | 10,540,961 | 527,034,550 | 10,083,726 | 95.66 |
| CCD3 | 10,826,048 | 541,302,400 | 10,326,227 | 95.38 |
| MT1 | 10,863,358 | 543,167,900 | 10,373,712 | 95.49 |
| MT2 | 12,482,706 | 624,135,300 | 11,913,708 | 95.44 |
| MT3 | 11,527,072 | 576,353,600 | 10,926,627 | 94.79 |
