# Supplementary material for: Transcriptome profiling of microRNAs reveals potential mechanisms of manual therapy alleviating neuropathic pain through microRNA-547-3p-mediated Map4k4/NF-κb signaling pathway
Source: J Neuroinflammation. 2022 Sep 1;19:211. doi: 10.1186/s12974-022-02568-x (PMC9434879; doi:10.1186/s12974-022-02568-x)
Supplement: Supplementary file 4 — Additional file 4. The the number of predicted target genes of differentially expressed miRNAs (CCD-sham). [file 12974_2022_2568_MOESM4_ESM.docx]

**The the number of predicted target genes of differentially expressed miRNAs (CCD-sham)**

| NO. | MiRNA | MiRDB | MiRTarBase | MiRWalk | TargetScan | TargetGeneNumber |
| --- | --- | --- | --- | --- | --- | --- |
| 1 | miR-375-3p | 111 | 3 | 2491 | 2255 | 86 |
| 2 | miR-449a-5p | 421 | 0 | 4808 | 389 | 45 |
| 3 | miR-21-5p | 173 | 12 | 2772 | 1793 | 147 |
| 4 | miR-135a-5p | 321 | 0 | 5018 | 2624 | 229 |
| 5 | miR-34c-3p | 121 | 0 | 2820 | 612 | 39 |
| 6 | miR-31a-5p | 230 | 3 | 4747 | 2835 | 187 |
| 7 | miR-206-3p | 405 | 5 | 4337 | 270 | 32 |
| 8 | miR-34c-5p | 386 | 2 | 4897 | 88 | 15 |
| 9 | miR-144-5p | 96 | 0 | 2354 | 1303 | 75 |
| 10 | miR-130b-5p | 334 | 0 | 5564 | 3938 | 258 |
| 11 | miR-3559-5p | 455 | 0 | 4833 | 3754 | 335 |
| 12 | miR-34b-5p | 387 | 0 | 4892 | 1170 | 135 |
| 13 | miR-133a-5p | 194 | 0 | 4477 | 2537 | 153 |
| 14 | miR-142-5p | 399 | 1 | 4444 | 3398 | 320 |
| 15 | miR-1-3p | 403 | 19 | 4549 | 2214 | 263 |
| 16 | miR-542-3p | 330 | 0 | 4478 | 3558 | 247 |
| 17 | miR-296-5p | 157 | 0 | 3795 | 2040 | 113 |
| 18 | miR-20a-5p | 552 | 0 | 4577 | 247 | 62 |
| 19 | miR-451-5p | 20 | 1 | 1047 | 528 | 13 |
| 20 | miR-96-5p | 419 | 1 | 4128 | 2850 | 351 |
| 21 | miR-183 | 42 | 0 | 2630 | 841 | 33 |
| 22 | miR-547-3p | 216 | 0 | 3212 | 2120 | 172 |


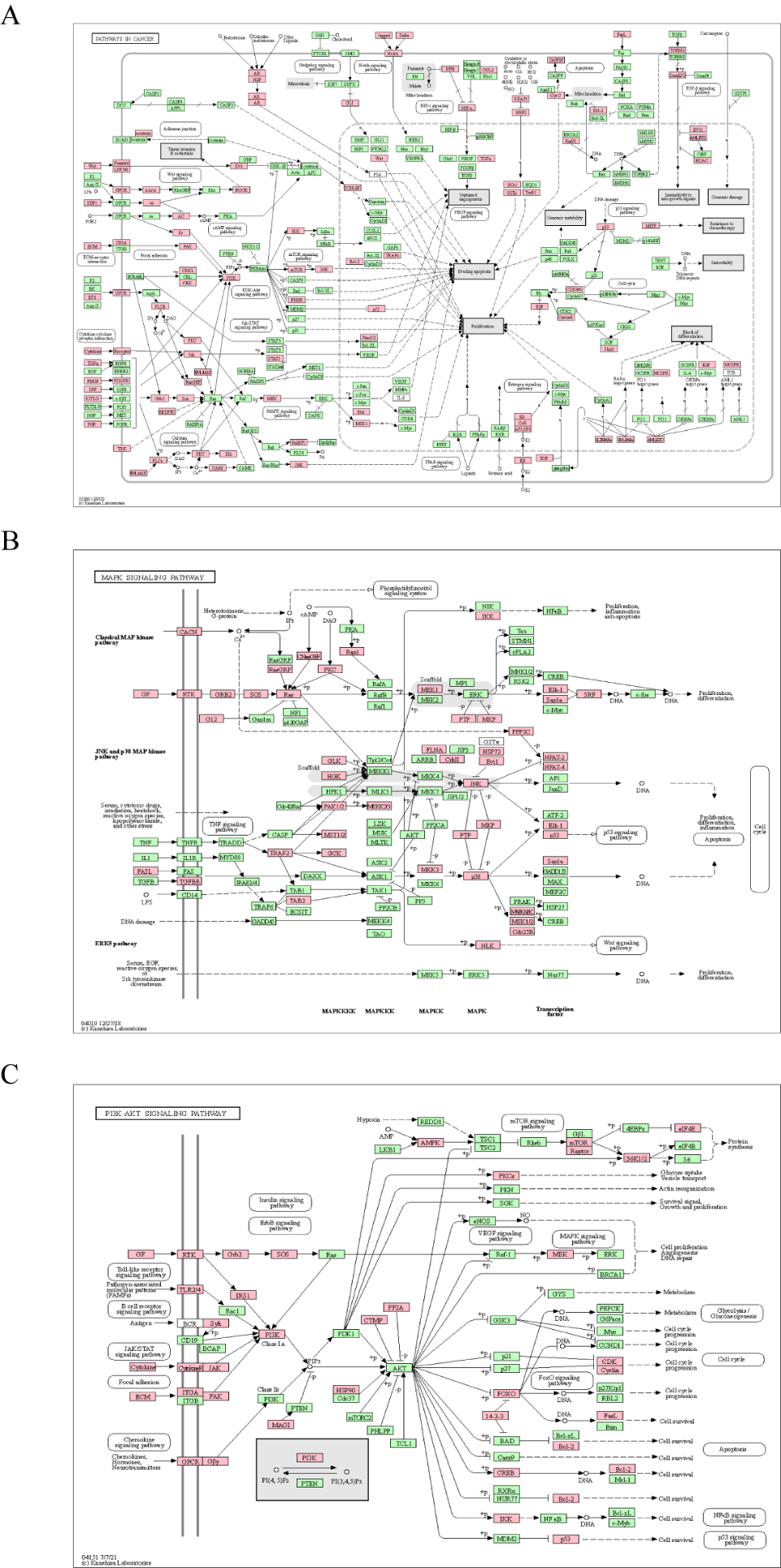


**The specific action modes of the predicted target genes (CCD-sham)**
