# Supplementary material for: Transcriptome profiling of microRNAs reveals potential mechanisms of manual therapy alleviating neuropathic pain through microRNA-547-3p-mediated Map4k4/NF-κb signaling pathway
Source: J Neuroinflammation. 2022 Sep 1;19:211. doi: 10.1186/s12974-022-02568-x (PMC9434879; doi:10.1186/s12974-022-02568-x)
Supplement: Supplementary file 5 — Additional file 5. The the number of predicted target genes of differentially expressed miRNAs (MT-CCD). [file 12974_2022_2568_MOESM5_ESM.docx]

**The the number of predicted target genes of differentially expressed miRNAs (MT-CCD)**

| NO. | MiRNA | MiRDB | MiRTarBase | MiRWalk | TargetScan | TargetGeneNumber |
| --- | --- | --- | --- | --- | --- | --- |
| 1 | miR-547-3p | 216 | 0 | 3212 | 2120 | 172 |
| 2 | miR-509-3p | 109 | 0 | 2730 | 1248 | 80 |
| 3 | miR-122-5p | 167 | 0 | 4569 | 2365 | 132 |
| 4 | miR-1247-5p | 26 | 0 | 0 | 654 | 0 |
| 5 | miR-493-3p | 191 | 0 | 4168 | 2341 | 135 |
| 6 | miR-539-5p | 407 | 0 | 5735 | 4362 | 314 |
| 7 | miR-504 | 169 | 0 | 3421 | 2612 | 128 |
| 8 | miR-183 | 42 | 0 | 2630 | 841 | 33 |
| 9 | miR-409a-3p | 86 | 1 | 3086 | 1547 | 54 |
| 10 | miR-211-5p | 448 | 0 | 4506 | 756 | 91 |
| 11 | miR-196a-5p | 117 | 0 | 3845 | 1161 | 60 |
| 12 | miR-219a-2-3p | 109 | 0 | 3295 | 1743 | 84 |
| 13 | miR-34c-5p | 386 | 2 | 4897 | 88 | 15 |
| 14 | miR-135a-5p | 321 | 0 | 5018 | 2624 | 229 |
| 15 | miR-34c-3p | 121 | 0 | 2820 | 612 | 39 |
| 16 | miR-449a-5p | 421 | 0 | 4808 | 389 | 45 |
| 17 | miR-375-3p | 111 | 3 | 2491 | 2255 | 86 |
| 18 | miR-879-5p | 196 | 0 | 4367 | 3083 | 148 |
| 19 | miR-743b-5p | 289 | 0 | 4573 | 2908 | 218 |


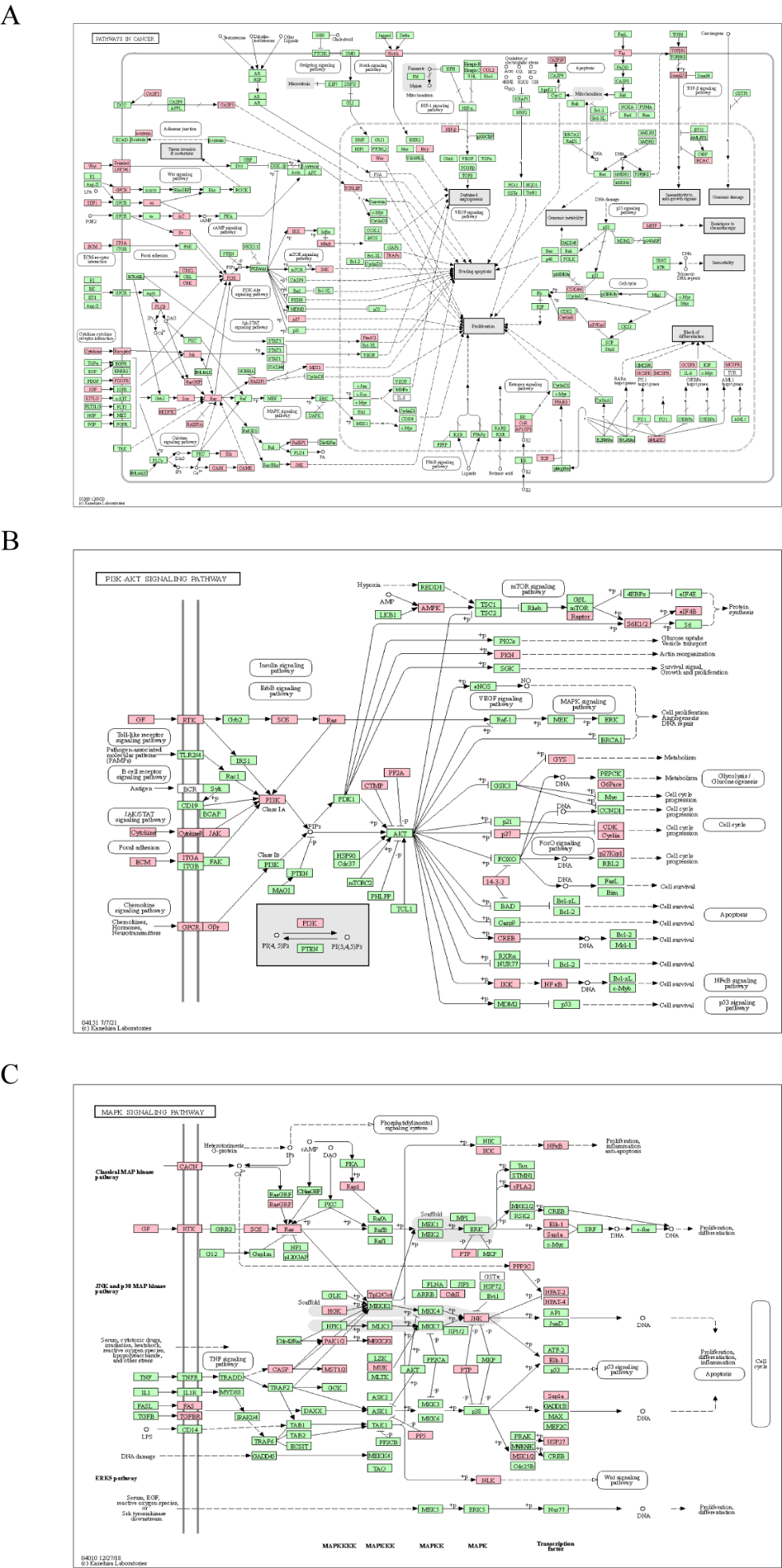


**The specific action modes of the predicted target genes (MT-CCD)**
